# The rare sugar d-allose acts as a triggering molecule of rice defence via ROS generation

**DOI:** 10.1093/jxb/ert282

**Published:** 2013-09-07

**Authors:** Akihito Kano, Takeshi Fukumoto, Kouhei Ohtani, Akihide Yoshihara, Toshiaki Ohara, Shigeyuki Tajima, Ken Izumori, Keiji Tanaka, Takeo Ohkouchi, Yutaka Ishida, Yoko Nishizawa, Kazuya Ichimura, Yasuomi Tada, Kenji Gomi, Kazuya Akimitsu

**Affiliations:** ^1^Faculty of Agriculture, Rare Sugar Research Center, and Gene Research Center, Kagawa University, Miki, Kagawa 761-0795, Japan; ^2^Mitsui Chemicals Agro Inc., Yasu, Shiga 520-2342, Japan; ^3^Shikoku Research Institute Inc., Yashima-nishi, Takamatsu 761-0192, Japan; ^4^National Institute of Agrobiological Sciences, Tsukuba 305-8602, Japan

**Keywords:** d-Allose, d-glucose 6-phosphate dehydrogenase, hexokinase, NADPH oxidase, *Oryza sativa* L, rare sugar.

## Abstract

Only d-allose, among various rare monosaccharides tested, induced resistance to *Xanthomonas oryzae* pv. *oryzae* in susceptible rice leaves with defence responses: reactive oxygen species, lesion mimic formation, and PR-protein gene expression. These responses were suppressed by ascorbic acid or diphenylene iodonium. Transgenic rice plants overexpressing *OsrbohC*, encoding NADPH oxidase, were enhanced in sensitivity to d-allose. d-Allose-mediated defence responses were suppressed by the presence of a hexokinase inhibitor. 6-Deoxy-d-allose, a structural derivative of d-allose unable to be phosphorylated, did not confer resistance. Transgenic rice plants expressing *Escherichia coli AlsK* encoding d-allose kinase to increase d-allose 6-phosphate synthesis were more sensitive to d-allose, but *E. coli AlsI* encoding d-allose 6-phosphate isomerase expression to decrease d-allose 6-phosphate reduced sensitivity. A d-glucose 6-phosphate dehydrogenase-defective mutant was also less sensitive, and *OsG6PDH1* complementation restored full sensitivity. These results reveal that a monosaccharide, d-allose, induces rice resistance to *X. oryzae* pv. *oryzae* by activating NADPH oxidase through the activity of d-glucose 6-phosphate dehydrogenase, initiated by hexokinase-mediated conversion of d-allose to d-allose 6-phosphate, and treatment with d-allose might prove to be useful for reducing disease development in rice.

## Introduction

Rare sugars are a group of ~50 monosaccharides that are present in very low amounts in the natural world (Izumori, 2002, 2006). Studies of rare sugars were limited by a lack of methods to produce these sugars on a bulk scale until the development of methodology for rare sugar production by Izumori’s group (Izumori, 2002, 2006). Recently, biological functions and metabolic pathways of one of these rare sugars, d-allose, for several organisms have been reported. In animals, d-allose can have an immunosuppressive effect (Hossain *et al.*, 2000) and a protective effect against liver damage (Hossain *et al.*, 2003). It can also inhibit cancer cell proliferation and production of reactive oxygen species (ROS) in neutrophils (Murata *et al.*, 2003; Sui *et al.*, 2005). Hamster fibroblasts form d-allose 6-phosphate (A6P) from d-allose, indicating that d-allose is transported and internally converted (Ullrey and Kalckar, 1991). In microbes, hexokinases of yeast and *Thermus caldophilus* can phosphorylate several monosaccharides including d-allose (Chenault *et al.*, 1997; Bae *et al.*, 2005). In *Aerobacter aerogenes* and *Escherichia coli*, d-allose is incorporated into the glycolytic pathway through conversion to d-fructose 6-phosphate (F6P) (Gibbins and Simpson, 1964; Kim *et al.*, 1997). Although d-allose has been detected in tissues of some plants (Perold *et al.*, 1973; Chari *et al.*, 1981; Jensen *et al.*, 1981; Weckwerth *et al.*, 2004), its function and metabolism in plants have not been well understood.

Recently, it was demonstrated in rice that application of d-allose inhibits the gibberellin signal transduction pathway downstream of the SLR1 protein step; thus, gibberellin-dependent responses, such as growth of seedlings and elongation of the second leaf sheath, were inhibited (Fukumoto *et al*., 2011, 2013). Because defence-related genes were up-regulated after d-allose treatment in an expression analysis using a rice microarray (Kano *et al.*, 2010) and in a quantitative reverse transcription–PCR (qRT–PCR) analysis of *Arabidopsis* (Narusaka *et al.*, 2009), treatment with d-allose might prove to be useful for reducing disease development (Kano *et al.*, 2010), but its mode(s) of action in defence induction has not been elucidated. In the present study, the discovery of the mechanism and function of d-allose in plant defence induction is described; phosphorylation of d-allose to A6P in d-allose-treated rice is essential to induce defence with lesion mimic formation initiated by the generation of ROS by NADPH oxidase, which is activated by NADPH supplied from d-glucose 6-phosphate dehydrogenase (G6PDH).

## Materials and methods

### Chemicals

Rare sugars and their derivatives (Supplementary Fig. S1 available at *JXB* online) with respective purities of 100% were prepared by the Rare Sugar Research Center at Kagawa University using methods described previously (Izumori, 2002, 2006). Common sugars, enzymes, and other reagents used in buffers, solvents, and reaction mixtures described in the respective sections were purchased from Wako (Tokyo, Japan) unless noted otherwise.

### Plant materials, sugar treatments, and bacterial inoculation

Rice plants (*Oryza sativa* L.) cv. Nipponbare were used as the wild type (WT) in the respective experiments. G6PDH1 mutants, selected from a search of the *Tos17* rice mutant database (Miyao *et al.*, 2003) (http://tos.nias.affrc.go.jp/), were obtained from the National Institute of Agrobiological Sciences (NIAS), Japan. WT, mutants overexpressing target genes (*OsrbohC*, *Alsk*, or *AlsI*), and *Tos17* mutants were grown to the six-leaf stage at 25 °C (14h light/10h dark) in plastic pots (9cm diameter×9cm height) with a small hole (1cm diameter) at the bottom to absorb water from a tray (20×14×7cm) containing 1 litre of water (Kano *et al*., 2010, 2011). Plants were then placed for 2 d on a tray containing either 1 litre of water or a sugar solution to observe lesion mimic formation and to measure lesion lengths. When ascorbic acid (AsA) or *N*-acetyl-d-glucosamine (GlcNAc) was used, 5mM AsA or 5mM GlcNAc was added to water with/or without 5mM d-allose. Plants for 3,3′-diaminobenzidine (DAB) staining and phosphorylated sugar detection were incubated with sugars and/or chemicals for 24h. For observation of lesion mimics, plants were placed in another tray with only water for 3 d after the 2 d sugar treatment. For the measurement of lesion length, plants were inoculated with a virulent race of *Xanthomonas oryzae* pv. *oryzae* (*Xoo*) (strain T7174) (~1×10^6^ CFU ml^–1^) after the 2 d sugar treatment, then incubated with water for 10 d as described previously (Kano *et al*., 2010, 2011).

### Chemical treatment for rice cut leaves

To visualize H_2_O_2_ accumulation, leaf tissues were stained with DAB (Sigma, St Louis, USA) as described by Torres *et al.* (2005). After treatment with 5mM d-allose alone or with 5mM AsA, 5mM GlcNAc, 25 µM diphenylene iodonium (DPI), or 25mM Na_3_PO_4_ for 24h, fully opened fifth leaf blades were immediately vacuum-infiltrated with 0.1% (w/v) DAB solution containing 0.1% (v/v) Triton X-100 and kept in the dark overnight. Coloured leaves were photographed after overnight destaining of excess DAB in ethanol/chloroform (4:1, v/v).

DPI was dissolved in 0.1% (v/v) dimethylsulphoxide (DMSO), and Na_3_PO_4_ was dissolved in distilled water and the solution was neutralized. Fifth leaf blades were removed from rice plants, and cut ends were placed in a solution of either 0.1% DMSO, 25 µM DPI (containing 0.1% DMSO), a mixture of 0.1% DMSO and 5mM d-allose, a mixture of 25 µM DPI, 25mM Na_3_PO_4_, and 5mM d-allose, or a mixture of 25mM Na_3_PO_4_ and 5mM d-allose, at 25 °C for 24h, before DAB staining.

### Detection of sugars and phosphorylated sugars by HPLC using ABEE labelling

The *p*-aminobenzoic acid ethyl ester (ABEE) labelling was performed as described by Yasuno *et al.* (1999) with modifications. Sugar-treated rice leaves (100mg) were ground in liquid nitrogen with a mortar and pestle. The powder-like tissues were mixed with 500 µl of extraction buffer (30mM potassium phosphate buffer, pH 7.6, containing 1mM EDTA), and centrifuged at 13 000rpm for 10min at 4 °C. The supernatant was passed through an Ultrafree-MC centrifugal filter unit (Millipore, Billerica, MA, USA) (0.22 µm). In the case of phosphatase treatment, the extracts (44 µl) were passed through the filter unit, then mixed with 1 µl (20U) of alkaline phosphatase (Takara, Shiga, Japan) and 5 µl of 10× buffer (in the enzyme kit), and incubated at 37 °C for 1h. For the recombinant enzyme assay, the reaction mixtures with the respective sugar substrate were passed through the filter units.

A 10 µl sample as prepared above was added to 40 µl of ABEE reagent solution (J-Oil Mills, Tokyo, Japan) with borane–pyridine complex (in the kit) and heated at 80 °C for 1h as per the manufacturer’s instructions. After the mixture cooled to room temperature, 200 µl each of distilled water and chloroform were added. After centrifugation of the mixture at 3000rpm for 5min, the upper aqueous layer was used for high-performance liquid chromatography (HPLC).

The layer containing ABEE-labelled sugars (10 µl) was analysed with an HPLC system (Prominence; Shimadzu, Kyoto, Japan) using an Xbridge C18 column (4.6mm ID×250mm) (Waters, Milford, MA, USA). A 50min separation at a flow rate of 1.0ml min^–1^ at 30 °C with a running solvent system of 0.2mM of potassium borate buffer (pH 8.9)/acetonitrile (93/7) was followed by a 20min wash with 0.02% trifluoroacetic acid/acetonitrile (50/50) and equilibration for 15min with the running solvent. The peaks were monitored with the fluorescence detector (RF-10A XL, Shimadzu) with emission of 360nm and excitation of 305nm.

### Cloning strategies

The coding region of *AlsK* and *AlsI* was amplified from *Escherichia coli* JM109 DNA by PCR using specific primers (Supplementary Table S1 at *JXB* online). The coding region of *OsHXK5*, *OsHXK6*, *OsrbohC*, *OsG6PDH1*, and *OsG6PDH2* was amplified from the respective cDNA clones that were provided by the Rice Genome Resource Center, Japan, using PCR and specific primers (Supplementary Table S1). The signal peptide regions of *OsHXK5* (135bp after the initiation codon) and *OsHXK6* (129bp after the initiation codon) were excluded. The DNA fragments were inserted into the pBI333-EN4 vector (Nishizawa *et al.*, 1999), pET32 vector (Novagen, Frankfurter, Germany), or pUC18-sGFP vector (Niwa *et al.*, 1999).

### Recombinant enzyme production and purification

The DNA fragment containing the coding region of *OsHXK5*, *OsHXK6*, *AlsK*, *OsG6PDH1*, or *OsG6PDH2* was subcloned in-frame into the pET32 vector (Novagen), and overexpressed in *E. coli* SoluBL21 (Genlantis, San Diego, CA, USA) according to the manufacturer’s instructions. The recombinant proteins were purified using a HisTrap HP column (GE Healthcare, Wauwatosa, WI, USA) as per the manufacturer’s instructions and dialysed against 0.2M TRIS-HCl buffer (pH 7.6) containing MgCl_2_ (5mM).

### Kinase assays

d-Glucose kinase activity of OsHXKs was measured spectrophotometrically at 340nm by coupling production of d-glucose 6-phosphate (G6P) to reduction of NADP via G6PDH reaction as described by Miller and Raines (2005). Reaction mixtures with 0.05–10mM d-glucose contained 0.2M TRIS (pH 7.6), NADP^+^ (0.5mM), dithiothreitol (DTT; 1mM), ATP (25mM), MgCl_2_ (5mM), and G6PDH (7.5U). d-Allose kinase activity of OsHXKs was determined spectrophotometrically (340nm) at 25 °C by coupling production of ADP to oxidation of NADH via pyruvate kinase (15U) (Oriental Yeast, Tokyo, Japan) and lactate dehydrogenase (25U) (Oriental Yeast) reactions as described by Miller and Raines (2005). Reaction mixtures with 0.1mM to 1M d-allose contained 0.2M TRIS (pH 7.6), NADH (0.5mM), DTT (1mM), phosphoenolpyruvate (5mM), ATP (25mM), MgCl_2_ (5mM), and KCl (5mM).

### Recombinant G6PDH assays

The activity of recombinant rice G6PDHs was measured spectrophotometrically (340nm) at 25 °C by detecting NADP reduction via G6PDH reaction, which is coupled with G6P production, as described by Wakao and Benning (2005). Reaction mixtures with 0.01–10mM G6P contained 0.2M TRIS (pH 7.6), NADP^+^ (0.01–10mM), and MgCl_2_ (5mM). DTT (10mM) was incubated with the reaction mixture for 1h to test its activity.

### G6PDH activity determination in protein extracts from rice leaves

Protein, extracted from rice leaves as described by Gibon *et al.* (2004), was added to dehydrogenase assay buffer (50mM TRIS-HCl, 5mM MgCl_2_, 0.5mM G6P, 1mM 6-phosphogluconate, 1mM NADP^+^, pH 7.6), and 6-phosphogluconate dehydrogenase (6PGD) assay buffer (50mM TRIS-HCl, 5mM MgCl_2_, 1mM 6-phosphogluconate, and 1mM NADP^+^, pH 7.6). The reduction of NADP^+^ to NADPH was assessed by absorbance change at 340nm. G6PDH activity was calculated as dehydrogenase activity minus 6PGD activity (Liu *et al.*, 2007).

### Rice transformation

The binary vector pBI333-EN4 (Nishizawa *et al.*, 1999) containing the target overexpression or complementation genes were introduced into *Agrobacterium tumefaciens* EHA101 by electroporation (Shen and Forde, 1989). Rice was transformed as described by Hiei *et al.* (1994). Second-generation plants were used for *Xoo* inoculation or various tests to determine the effect on d-allose-induced responses described in other sections.

### RT–PCR and qRT–PCR analysis

Total RNA was isolated from rice leaves with Trizol Reagent Kit (Invitrogen, San Diego, CA, USA). RT–PCR was performed with OneStep RT-PCR Kit (Qiagen, Hilden, Germany) for transgenic and *Tos17*-inserted rice with gene-specific primers (Supplementary Table S1 at *JXB* online) as described previously (Gomi *et al.*, 2010). For qRT–PCR, reverse transcription was performed using the Prime Script RT Reagent Kit (Takara) with specific primers (Supplementary Table S1) by a Thermal Cycler Dice TP800 (Takara) and SYBR Premix Ex Taq Mixture (Takara). The transcript level was normalized by comparison with actin (AK060893), and the obtained data were analysed as described previously (Kano *et al*., 2010, 2011).

## Results

### Effect of rare sugars on induction of rice disease resistance to *X. oryzae* pv. *oryzae*

**Fig. 1.**
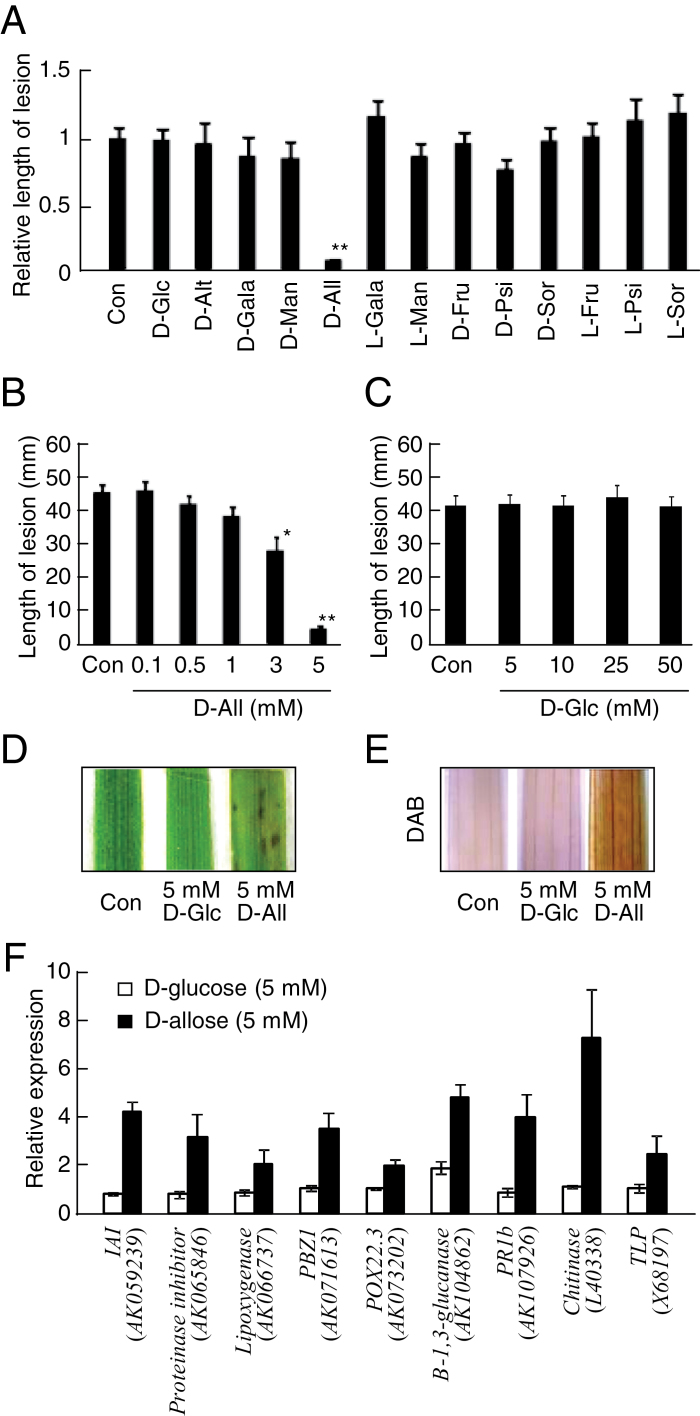
Rare sugar effects on induction of rice resistance to *Xoo*. Mean lesion length (±SE, *n*=12) on leaves treated for 2 d before *Xoo* inoculation with (A) 5mM sugars, (B) 0.1–5mM d-allose, or (C) 5–50mM d-glucose. Lesion development 10 d after *Xoo* inoculation is indicated as values relative to control (A) or lesion lengths (B, C) (*P < 0.05, **P < 0.01). (D) Lesion mimic development on leaves 3 d after a 2 d treatment with 5mM d-glucose or d-allose. (E) DAB detection of H_2_O_2_ accumulation in leaves after 24h treatment with 5mM d-glucose or d-allose. (F) Expression of defence-related genes at 2 d after treatment with 5mM d-allose or d-glucose. Fold (±SE, *n*=4) expression relative to control (no sugar) is shown. The following abbreviations are used in all figures and tables: d-Glc, d-glucose; d-Alt, d-altrose; d-Gala, d-galactose; d-Man, d-mannose; d-All, d-allose; l-Gala,l-galactose; l-Man, l-mannose; d-Fru, d-fructose; d-Psi, d-psicose; d-Sor, d-sorbose; l-Fru, l-fructose; l-Psi, l-psicose; l-Sor, l-sorbose; Con, control; and DAB, 3,3′-diaminobenzidine. (This figure is available in colour at *JXB* online.)

**Fig. 2.**
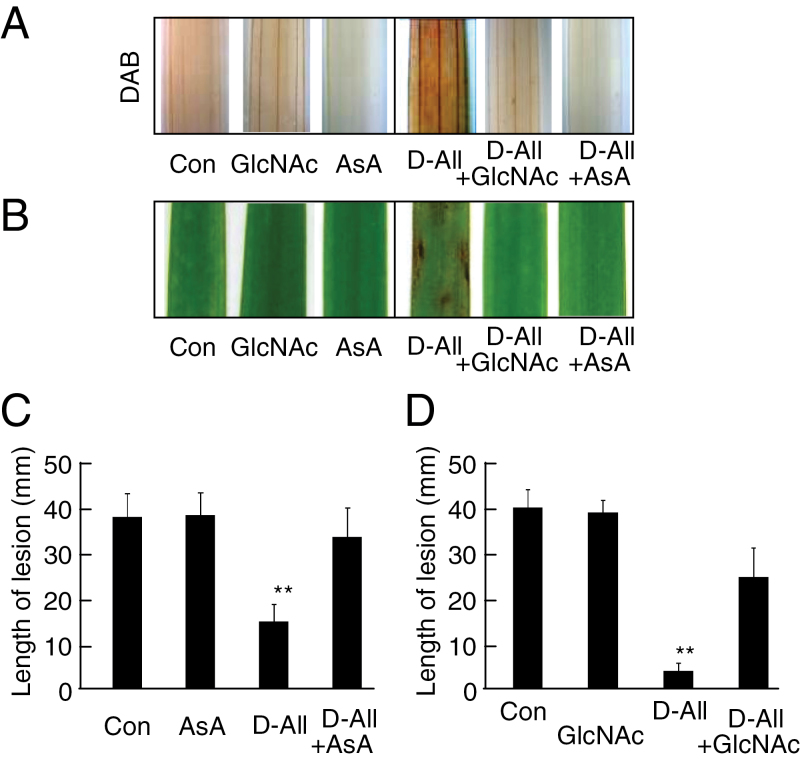
Ascorbic acid (AsA) or *N*-acetyl-d-glucosamine (GlcNAc) effect on d-allose-induced resistance. (A) DAB detection of H_2_O_2_ accumulation in leaves after 24h treatment with 5mM AsA or GlcNAc with or without 5mM d-allose. (B) Lesion mimic development on leaves 3 d after a 2 d treatment with 5mM AsA or GlcNAc with or without 5mM d-allose. (C and D) Mean lesion length (±SE, *n*=12) on leaves pre-treated with 5mM AsA (C) or GlcNAc (D) with or without 5mM d-allose (**P < 0.01). (This figure is available in colour at *JXB* online.)

Nine rare sugars and four common sugars (Supplementary Fig. S1 at *JXB* online) were tested for their ability to induce disease resistance after sugar-treated rice leaves were inoculated with *Xoo* (Fig. 1). The mean length of lesions was only inhibited after treatment with 5mM d-allose (Fig. 1A), but did not differ significantly after mock or other sugar treatments (Fig. 1A). Disease resistance caused by the d-allose treatment was induced in a dose-dependent manner, with the reduction of lesion development starting at 3mM, while d-glucose produced no inhibition even at 50mM (Fig. 1B, C). The d-allose-specific induction of resistance to *Xoo* was associated with formation of lesion mimics on the rice leaves (Fig. 1D). Since the lesion mimic after a hypersensitive response is often induced by production of ROS and is associated with induction of disease resistance to *Xoo* in rice (Yin *et al.*, 2000; Ono *et al.*, 2001; Torres *et al.*, 2005), hydrogen peroxide (H_2_O_2_) production was monitored as an indicator of ROS generation by staining leaf tissues with DAB after the d-allose treatment (Fig. 1E). The level of H_2_O_2_ was significantly higher in d-allose-treated rice leaves than in d-glucose-treated or mock-treated leaves (Fig. 1E), and expression of defence-related PR-protein genes was also induced in the d-allose-treated rice leaves (Fig. 1F). d-Allose had no visible effect on growth of *Xoo* (Supplementary Fig. S2). The d-allose-mediated induction of ROS accumulation, lesion mimic formation, and resistance to *Xoo* was suppressed by simultaneous treatment with AsA, a scavenger of ROS (Fig. 2A–C).

### *OsrbohC* is involved in d-allose-induced resistance to *Xoo*

**Fig. 3.**
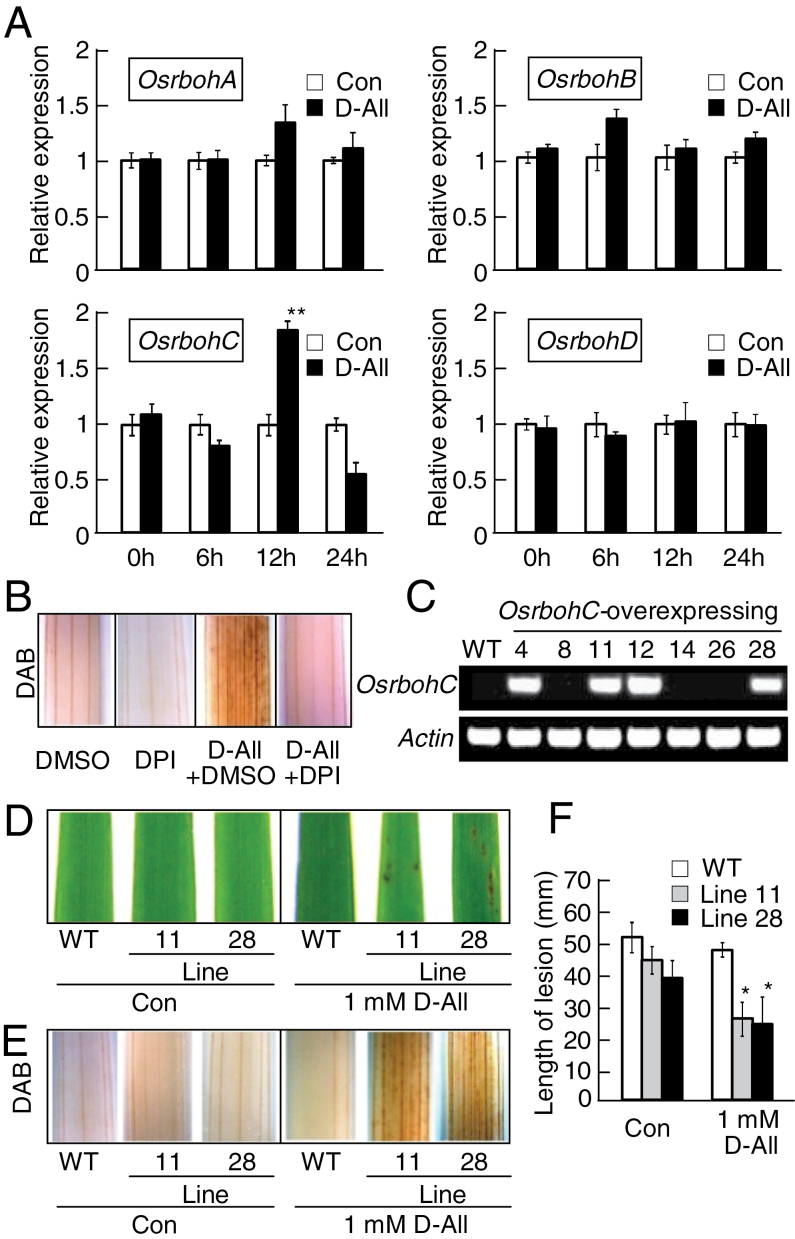
OsrbohC is involved in d-allose-induced resistance to *Xoo*. (A) *Osrboh* gene expression in leaves at 0–24h after treatment with 5mM d-allose or no sugar (control) was calculated as values (±SE, *n*=3) relative to control. Accessions: *OsrbohA* (AK103747), *OsrbohB* (AK065117), *OsrbohC* (AK120905), and *OsrbohD* (AK072353) (**P < 0.01). (B) Effect of diphenylene iodonium (DPI) treatment on d-allose-induced H_2_O_2_ accumulation in leaves. (C) RT–PCR detection of *OsrbohC* and *actin* gene expressions in leaves from WT and *OsrbohC*-overexpressing rice. (D) Lesion mimic development in leaves from WT and *OsrbohC*-overexpressing rice 3 d after a 2 d treatment with 1mM d-allose. (E) DAB detection of H_2_O_2_ accumulation after 24h treatment with 1mM d-allose in leaves from WT and *OsrbohC*-overexpressing rice. (F) Mean lesion lengths (±SE, *n*=8) 10 d after *Xoo* inoculation in leaves pre-treated for 2 d with 1mM d-allose from WT and *OsrbohC*-overexpressing rice (*P < 0.05). (This figure is available in colour at *JXB* online.)

d-Allose induced ROS accumulation (Figs 1E, 2A). NADPH oxidase, encoded by members of the *Respiratory burst oxidase homolog* (*Rboh*) gene family, is a known generator of ROS during the defence response of many plants including rice (Doke, 1985; Torres *et al.*, 2005; Sagi and Fluhr, 2006). Thus, induction patterns in d-allose-treated leaves of rice *Rboh* genes (*OsrbohA–OsrbohD*) were examined by qRT–PCR analysis (Fig. 3A). Quantitative analysis over time indicated that only *OsrbohC* was induced at 12h after d-allose treatment (Fig. 3A). Treatment with DPI, an NADPH oxidase inhibitor (Kawasaki *et al.*, 1999), inhibited the accumulation of H_2_O_2_ in d-allose-treated leaves (Fig. 3B).

To examine further the contribution of OsrbohC to ROS generation in d-allose-treated leaves, transgenic rice plants overexpressing *OsrbohC* were generated (Supplementary Fig. S3A at *JXB* online). Two-independent lines (lines 11 and 28) were selected from multiple transgenic rice plants expressing *OsrbohC* (Fig. 3C), and the second generation of these lines was tested further. The overexpression of *OsrbohC* did not influence growth or any visible phenotype of rice (Supplementary Fig. S3B), and the excess *OsrbohC* did not change the sensitivity to *Xoo* with/or without d-glucose treatment (Supplementary Fig. S3C).

When the *OsrbohC*-overexpressing plants were treated with even 1mM d-allose, lesion mimics formed on the leaves, but not on the treated WT (Fig. 3D). H_2_O_2_ generation was much stronger in leaves of transgenic plants treated with 1mM d-allose than in those of the WT (Fig. 3E). When the transgenic plants were inoculated with *Xoo*, blight lesions were significantly shorter on leaves of 1mM d-allose-treated transgenic plants than on those of the WT (Fig. 3F).

### Phosphorylation of d-allose at carbon 6 is important for rice resistance induction

**Fig. 4.**
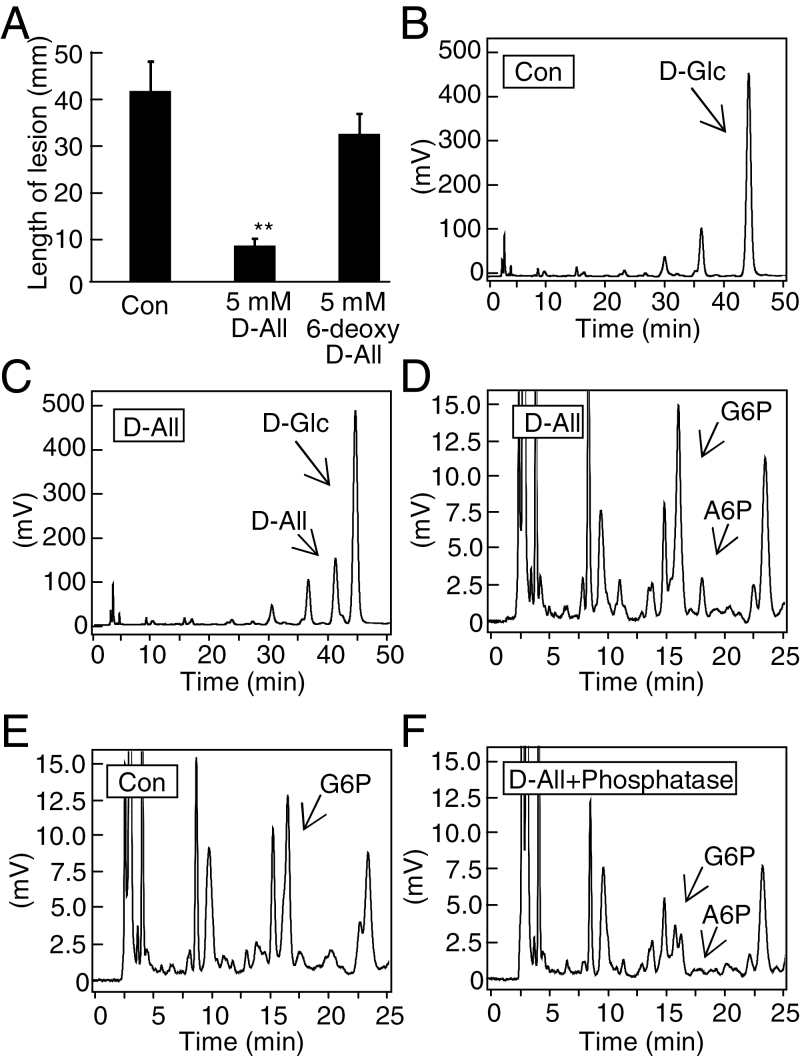
Detection of phosphorylated d-allose in d-allose-treated leaves. (A) Mean lesion length (±SE, *n*=12) 10 d after *Xoo* inoculation of leaves pre-treated for 2 d with 5mM d-allose or 6-deoxy-d-allose (**P < 0.01). (B–E) HPLC detection of ABEE-labelled monosaccharides (Yasuno *et al.*, 1999) in extracts from leaves treated with (C and D) or without d-allose (B and E) for 24h. (D) Close-up of the chart to show phosphorylated sugars of (C). (E) Close-up of (B). (F) Reduction of phosphorylated sugars by phosphatase addition in extracts from leaves treated with d-allose. A comparative scale view to (D) is shown. Calculated values of sugars in leaves were 852ng g FW^–1^ for d-allose and 2.7 µg g FW^–1^ for d-glucose in (C). The following abbreviations are used in all figures and tables: A6P, d-allose 6-phosphate; G6P, d-glucose 6-phosphate.

6-Deoxy-d-allose, a derivative of d-allose with a methyl group provided by conversion of a hydroxyl group to hydrogen on carbon 6 (Supplementary Fig. S1 at *JXB* online), did not confer resistance to *Xoo* (Fig. 4A). Since the hydroxyl group is often a phosphorylation site on sugars, HPLC was used to check for phosphorylated d-allose in d-allose-treated leaves. The major peak in extracts of mock-treated rice tissue was d-glucose (Fig. 4B), while a d-allose peak was detected in d-allose-treated leaves (Fig. 4C). In addition, a peak of A6P (retention time 17.5min) was detected in extracts from d-allose-treated rice leaves (Fig. 4D), but not from mock-treated leaves (Fig. 4E). Alkaline phosphatase addition to the extracts significantly reduced the peak size of A6P and G6P (Fig. 4F).

**Fig. 5.**
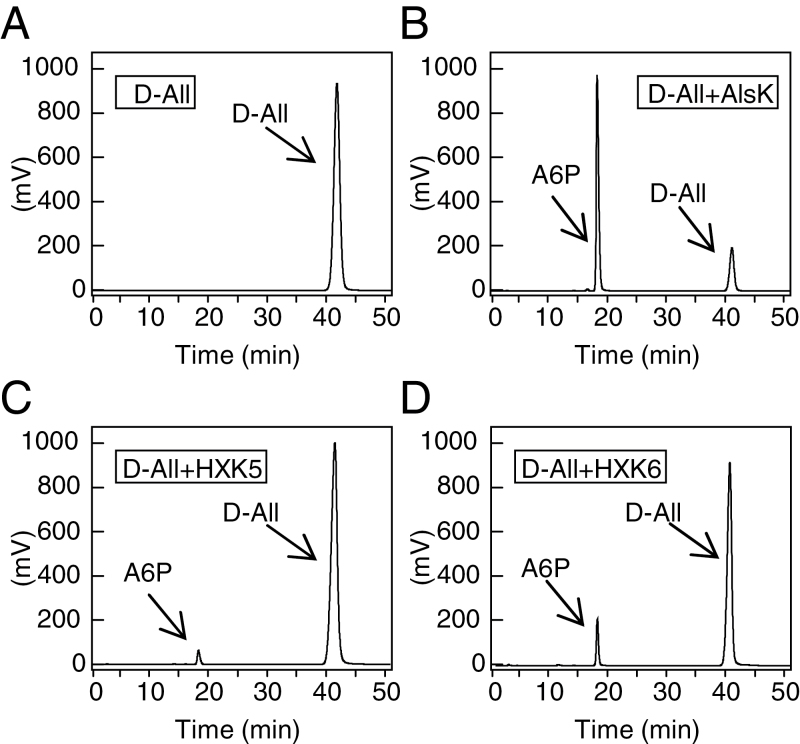
d-Allose phosphorylation by recombinant kinases. (A–D) Products of recombinant d-allose kinase (AlsK) and rice hexokinases (HXK5 and HXK6) reacted with 5mM d-allose were labelled by ABEE (Yasuno *et al.*, 1999) and determined by HPLC. (A) d-Allose alone without any recombinant enzyme. (B) AlsK reacted with d-allose. (C) HXK5 reacted with d-allose. (D) HXK6 reacted with d-allose.

Hexokinases of yeast and *T. caldophilus* use several sugars including d-allose as substrates (Chenault *et al.*, 1997; Bae *et al.*, 2005). A6P levels were thus measured after supplying d-allose as a substrate for two main rice hexokinases, OsHXK5 and OsHXK6 (Cho *et al.*, 2009) using their respective recombinants OsHXK5 and OsHXK6 (Fig. 5) or a recombinant d-allose kinase (AlsK) of *E. coli* (Miller and Raines, 2005) (Supplementary Fig. S4 at *JXB* online) as the positive control for A6P production (Fig. 5). *K*_m_ values of OsHXK5 and OsHXK6 for d-allose differed by two orders of magnitude from those for d-glucose, but the difference in affinity was lower than that of AlsK for d-glucose (Table 1) (Miller and Raines, 2005). Based on comparisons of the *k*_cat_/*K*_m_ values, enzymatic activity of AlsK for d-allose conversion to A6P was >100 times more efficient than that of OsHXK5 or OsHXK6, but both OsHXK5 and OsHXK6 were also highly active in converting d-allose to A6P (Fig. 5, Table 1). Moreover, d-allose-mediated induction of *Xoo* resistance was suppressed by treatment with GlcNAc, an inhibitor of HXK, as were ROS generation and subsequent lesion mimic development (Fig. 2A, B, D).

**Table 1.** Rice hexokinase OsHXK5 and OsHXK6 can use d-allose as substrate

Substrate	*k*_cat_ (s^–1^)	*K*_m_ (M)	*k*_cat_/*K*_m_ (M^–1^ s^–1^)	Reference
OsHXK5				
d-Glucose	205	1.9×10^–4^	1.1×10^6^	This study
d-Allose	13	3.8×10^–2^	3.4×10^2^	This study
OsHXK6				
d-Glucose	106.5	2.0×10^–4^	5.3×10^5^	This study
d-Allose	18	3.7×10^–2^	4.9×10^2^	This study
Allose kinase				
d-Glucose	1.5	1.0×10^–1^	1.5×10^1^	Miller and Raines (2005)
d-Allose	17	2.6×10^–4^	6.5×10^4^	Miller and Raines (2005)

Sugar kinase activity was determined by the methods of Miller and Raines (2005).

### Overexpression of *AlsK* increased sensitivity to d-allose in rice plants

**Fig. 6.**
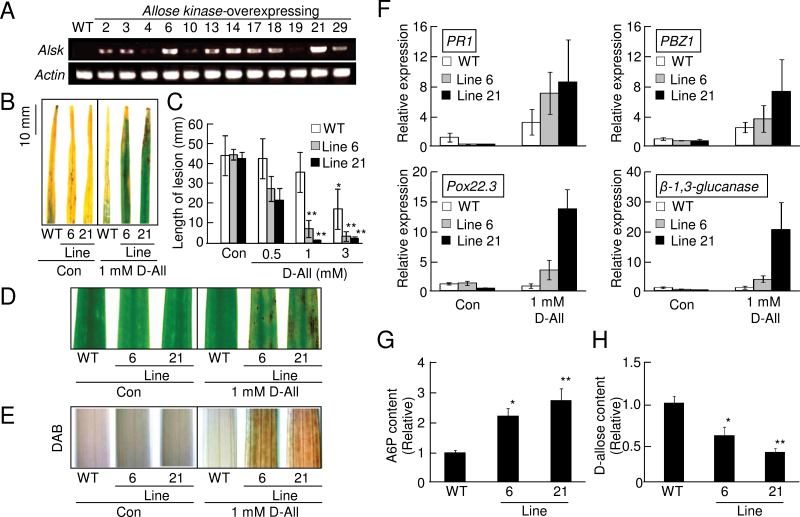
d-Allose sensitivity increased by *AlsK* overexpression in rice. (A) RT–PCR detection of *AlsK* and *actin* expression in leaves from WT and different lines of *AlsK*-overexpressing rice. (B) Typical lesion development 10 d after *Xoo* inoculation of WT and *AlsK*-overexpressing lines pre-treated with or without 1mM d-allose. (C) Mean lesion length (±SE, *n*=8) 10 d after *Xoo* inoculation in leaves pre-treated for 2 d with 0.5–3mM d-allose (**P < 0.01 compared with the WT without d-allose treatment). (D) Lesion mimic development in leaves from WT and *AlsK*-overexpressing rice at 3 d after a 2 d treatment with 1mM d-allose. (E) DAB detection of H_2_O_2_ accumulation at 24h after treatment with 1mM d-allose in leaves from WT and *AlsK*-overexpressing rice plants. (F) Expression of defence-related genes at 2 d after treatment with 1mM d-allose. Fold (±SE, *n*=4) expression relative to control (no sugar) is shown. Accession numbers are given in Fig. 1. (G, H) d-Allose 6-phosphate (A6P) (G) or d-allose (H) content detected by HPLC in leaves from WT and *AlsK*-overexpressing lines at 24h after treatment with 5mM d-allose. Values are relative (±SE, *n*=3) to the WT (*P < 0.05, **P < 0.01). The calculated value of d-allose content was 756ng g FW^–1^ in (H). (This figure is available in colour at *JXB* online.)

Since the phosphorylation of d-allose by *E. coli* AlsK was much more efficient than that by OsHXK5 and OsHXK6 (Table 1), transgenic rice plants were generated that constitutively expressed *E. coli AlsK* (Miller and Raines, 2005) (Supplementary Fig. S5A at *JXB* online) to enhance the efficiency of d-allose conversion to A6P. Two independent lines (lines 6 and 21) were selected among transgenic rice plants that were confirmed by RT–PCR to express *AlsK* (Fig. 6A), and the second generation of these lines was used for further experiments. Overexpression of *AlsK* did not influence growth or any visible trait of rice (Supplementary Fig. S5B), and excess AlsK did not change the sensitivity to *Xoo* with/or without d-glucose treatment (Supplementary Fig. S5C). There was also no significant difference in the ratios of inherent d-glucose and G6P contents between the *AlsK*-overexpressing plants and the WT (Supplementary Fig. S5D, E). However, when these rice plants were treated with even 1 mM d-allose, the blight lesions on the *AlsK*-expressing plants were significantly shorter than those on the WT (Fig. 6B, C). The enhanced resistance was associated with lesion mimic formation in the *AlsK*-expressing plants treated with 1mM d-allose (Fig. 6D), and H_2_O_2_ accumulation was also enhanced (Fig. 6E). Expression of PR-protein genes including probenazole-inducible protein (*PBZ1*), pathogenesis-related protein 1b (*PR1b*), peroxidase (*Pox22.3*), and *β-1,3-glucanase*, which are known to be induced strongly by >5mM d-allose in the WT (Fig. 1F), were significantly induced even by 1mM d-allose (Fig. 6F).

The HPLC peak area corresponding to A6P in both lines (6 and 21) of the d-allose-treated *AlsK*-overexpressing plants was significantly higher than in the WT (Fig. 6G; Supplementary Fig. S5G, I, K at *JXB* online), and those for d-allose were lower in the transgenic plants than in the WT (Fig. 6H; Supplementary Fig. S5F, H, J).

### Overexpression of *AlsI* decreased sensitivity to d-allose in rice plants

**Fig. 7.**
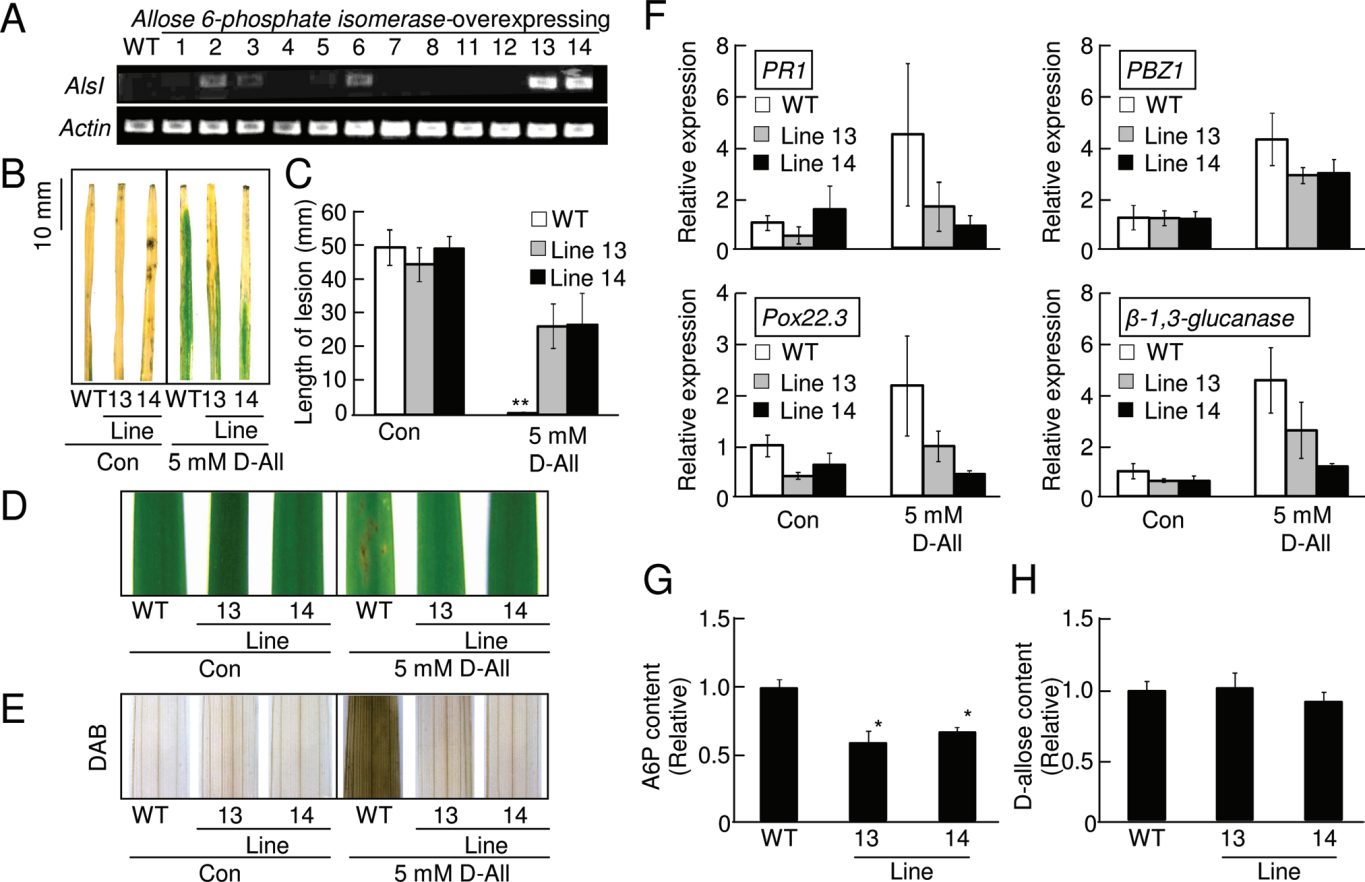
d-Allose sensitivity decreased by *AlsI* overexpression in rice. (A) RT–PCR detection of *AlsI* and *actin* expression in leaves from the WT and different lines of *AlsI*-overexpressing rice. (B) Typical lesion development at 10 d after *Xoo* inoculation of the WT and *AlsI*-overexpressing lines pre-treated with or without 5mM d-allose. (C) Mean lesion length (±SE, *n*=8) at 10 d after *Xoo* inoculation of leaves pre-treated for 2 d with 5mM d-allose (**P < 0.01 compared with the WT without d-allose treatment). (D) Lesion mimic development in leaves from WT and *AlsI*-overexpressing rice 3 d after a 2 d treatment with 5mM d-allose. (E) DAB detection of H_2_O_2_ accumulation 24h after treatment with 5mM d-allose in leaves from WT and *AlsI*-overexpressing plants. (F) Expression of defence-related genes at 2 d after treatment with 5mM d-allose. Fold (±SE, *n*=4) expression relative to the control (no sugar) is shown. (G, H) d-Allose 6-phosphate (A6P) (G) or d-allose (H) content detected by HPLC in leaves from the WT and *AlsI*-overexpressing lines at 24h after treatment with 5mM d-allose. Values are relative (±SE, *n*=3) to the WT (*P < 0.05). The calculated value of d-allose content was 823ng g FW^–1^ in (H). (This figure is available in colour at *JXB* online.)

Since *E. coli* d-allose 6-phosphate isomerases (AlsI) is known to convert A6P to d-psicose 6-phosphate (P6P) (Kim *et al.*, 1997) (Supplementary Fig. S4 at *JXB* online), transgenic rice plants constitutively expressing *E. coli AlsI* were generated (Supplementary Fig. S6A) to decrease A6P by conversion to P6P. Two independent lines (lines 13 and 14) expressing *AlsI* were selected (Fig. 7A), and the second generation of these lines was used for further experiments. Overexpression of *AlsI* did not affect growth or any visible trait of rice (Supplementary Fig. S6B). When these *AlsI*-expressing rice plants were treated with 5 mM d-allose and inoculated with *Xoo*, d-allose-induced resistance was reduced, and blight lesion formation was significantly increased (Fig. 7B, C). The reduced d-allose-induced resistance to *Xoo* in the *AlsI*-expressing plants was associated with reduced lesion mimic formation (Fig. 7D), accumulation of H_2_O_2_ (Fig. 7E), and expression of the PR-protein gene (Fig. 7F).

The HPLC peak area corresponding to A6P in both lines (13 and 14) of the d-allose-treated *AlsI*-overexpressing plants was significantly lower than in the WT (Fig. 7G; Supplementary Fig. S6D, F, H at *JXB* online), and those for d-allose did not change (Fig. 7H; Supplementary Fig. S6C, E, G).

### Reduced sensitivity to d-allose in the G6PDH-defective rice mutant

**Fig. 8.**
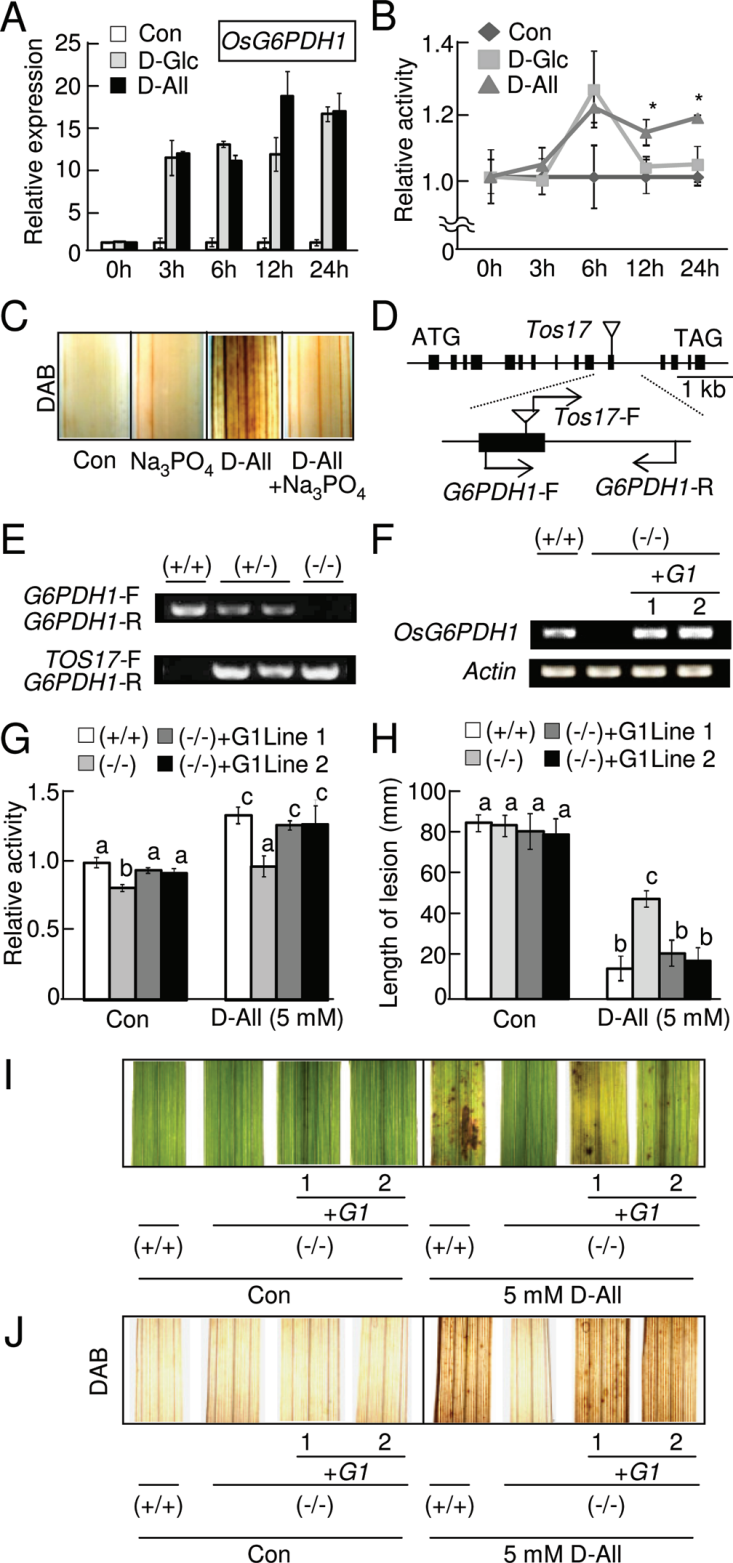
Role of OsG6PDH1 in the d-allose signalling pathway. (A) *OsG6PDH1* gene (accession no. AK073697) expression in leaves at 0–24h after treatment with 5mM d-allose or d-glucose, calculated relative (±SE, *n*=4) to the control (no sugar). (B) Total G6PDH activity in extracts from leaves at 0–24h after treatment with 5mM d-allose or d-glucose, calculated as values relative (±SE, *n*=4) to the control (no sugar) (*P < 0.05). The calculated value of the enzymatic activity for the control at 0h was 99 µmol NADPH min^–1^ g FW^–1^. (C) DAB detection of H_2_O_2_ accumulation at 24h after treatment with 5mM d-allose with or without a G6PDH inhibitor (Na_3_PO_4_). (D) Exon and intron organization of *OsG6PDH1* and the *Tos17* insertion site with locations of specific primers. Solid boxes and lines indicate exons and introns; triangles indicate the position of *Tos17* insertion. (E) Genotypic determination for wild homozygote (+/+), heterozygote (+/–), or homozygote for *Tos17* insertion (–/–) in the *Tos17* mutant line NC8489 by genomic PCR with primer pairs in (D). (F) *OsG6PDH1* transcript accumulations in the leaves by RT–PCR. Abbreviations in (F–J): (+/+) wild homozygote, (–/–) homozygote for *Tos17* insertion, or (+G1) two lines of the *OsG6PDH1* complementation mutant in *Tos17* mutant line NC8489, respectively. (G) Total G6PDH activity in extracts from leaves of (+/+), (–/–), or (+G1) at 0 or 24h after treatment with 5mM d-allose, calculated as values relative (±SE, *n*=4) to (+/+) (no sugar). Means with different letters are significantly different at *P* < 0.05. (H) Mean lesion length (±SE, *n*=8) 10 d after *Xoo* inoculation in leaves pre-treated for 2 d with or without 5mM d-allose. Means with different letters are significantly different at *P* < 0.05. (I) Lesion mimic development in leaves from (+/+), (–/–), or (+G1) at 3 d after a 2 d treatment with 5mM d-allose. (J) DAB detection of H_2_O_2_ accumulation at 24h after treatment with 5mM d-allose in leaves from (+/+), (–/–), or (+G1) rice plants. (This figure is available in colour at *JXB* online.)

In this study, it was found that rice hexokinases can catalyse the conversion of d-allose to A6P (Fig. 5, Table 1), which accumulates and leads to the induction of defence responses (Figs 2A, B, D, 4, 6, 7). Since the hexokinase product (G6P) from d-glucose can serve as the substrate of G6PDH, which can supply NADPH to NADPH oxidase for ROS generation (e.g. Scharte *et al.*, 2009; Gutpe *et al*., 2009; Spencer *et al.*, 2011), the involvement of G6PDH in d-allose signal transduction was examined. Among five genes encoding G6PDH in the rice genome (*OsG6PDH1–OsG6PDH5*), expression of *OsG6PDH1* (Fig. 8A), *OsG6PDH3*, and *OsG6PDH5* (Supplementary Fig. S7A at *JXB* online) was induced as soon as 3h after treatment with d-glucose or d-allose, and expression of *OsG6PDH1* at 12h (Fig. 8A) and of *OsG6PDH3* at 12h and 24h after d-allose treatment (Supplementary Fig. S7A) was higher than with d-glucose.

Phylogenic analyses of *OsG6PDH* genes against *Arabidopsis G6PD* genes encoding G6PDH predicted that OsG6PDH1 and OsG6PDH2 are in the cytoplasm (Supplementary Fig. S7B at *JXB* online) because *Arabidopsis* G6PD5 and G6PD6 in the same clade are cytoplasmic (Wakao *et al.*, 2008). There is no typical sorting signal present in OsG6PDH1 and OsG6PDH2, and green fluorescent protein (GFP)-tagged OsG6PDH1 and G6PDH2 in the bombarded tobacco leaf cells localized in the cytoplasm (Supplementary Fig. S7C).

Recombinant proteins of OsG6PDH1 and OsG6PDH2 had enzymatic activity with G6P and NADP^+^, and OsG6PDH1 had higher activity based on *k*_cat_/*K*_m_ values (Table 2). OsG6PDH1 and OsG6PDH2 suffered tight feedback inhibition by NADPH (Supplementary Table S2 at *JXB* online), and DTT did not affect activity with G6P (Supplementary Table S3) as described for *Arabidopsis* G6PDs (Wakao and Benning, 2005). Neither enzyme used A6P as a substrate (Supplementary Table S3). Interestingly, when total OsG6PDH activity was measured using protein extracts of rice leaf tissues at various times after either d-glucose or d-allose treatment, G6PDH activity had increased by 6h after d-glucose and d-allose treatments (Fig. 8B). However, activity dropped to the control level after 12h with d-glucose, but activity did not drop with d-allose even after 24h (Fig. 8B). Na_3_PO_4_, an inhibitor of G6PDH (Liu *et al.*, 2007), significantly reduced H_2_O_2_ accumulation in d-allose-treated leaves (Fig. 8C), also implicating G6PDH in d-allose signal transduction.

**Table 2.** Enzymatic profiles for OsG6PDH1 and OsG6PDH2 recombinant proteins using G6P as a kinetic parameter

Enzyme	*k*_cat_ (s^–1^)	*K*_m_ G6P (M)	*k*_cat_/*K*_m_ G6P (M^–1^ s^–1^)
G6PDH1	3.04	6.06×10^–4^	5.02×10^3^
G6PDH2	1.06	1.05×10^–3^	1.01×10^3^

Kinetic parameters were determined using a G6PDH-coupled assay for G6P (Wakao and Benning, 2005).

To examine further the role of cytosolic *OsG6PDH1* and *OsG6PDH2* in d-allose signalling, several retrotransposon *Tos17* insertion lines were obtained and line NC8489 was examined for an *OsG6PDH1* mutation. Several *Tos17*-insertion mutants for *OsG6PDH1* were found (Supplementary Fig. S8A at *JXB* online), but none for *OsG6PDH2*. Among mutant lines, *Tos17* was inserted at the target site in exon 11 in NC8489, which was then examined further (Fig. 8D; Supplementary Fig. S8A).

In the homozygous NC8489 line (–/–), *OsG6PDH1* was not amplified from genomic DNA (Fig. 8E) or mRNA (Fig. 8F). Total enzyme activity of G6PDHs in leaf extracts was reduced in the line with/or without 5mM d-allose (Fig. 8G), and d-allose-induced resistance to *Xoo* was lower than in the WT (+/+) (Fig. 8H). The reduced d-allose-induced resistance to *Xoo* in the line was associated with reductions of lesion mimic formation (Fig. 8I) and H_2_O_2_ accumulation (Fig. 8J). To confirm that this reduction in d-allose sensitivity was caused by the loss of *OsG6PDH1*, the intact *OsG6PDH1* gene was introduced into line NC8489 (–/–) for a complementation analysis. When the *OsG6PDH1* promoter region (2496bp) connected to *OsG6PDH1* (Supplementary Fig. S8B at *JXB* online) was introduced (+G1 line 1 and line 2) (Fig. 8F–J), transcription and protein function of *OsG6PDH1* was recovered in two independent complementation lines (+G1 line 1 and line 2) (Fig. 8F, G), and their sensitivities for d-allose were nearly equal to that of the WT (+/+) (Fig. 8H–J).

## Discussion

Natural oligosaccharides and salicylic acid are known as plant defence activators that induce PR-protein gene expression and defence responses in many different plants (e.g. Dixon and Lamb, 1990; Ebel and Cosio, 1994; Klessig and Malamy, 1994). Some monosaccharides, mainly d-glucose and d-fructose at high concentrations, can also regulate growth of higher plants (Rolland *et al.*, 2006); however, monosaccharides have never been reported to be deeply involved in the induction of a plant defence system. As far as is known, the present finding that d-allose induced a rice defence reaction against *Xoo* that included lesion mimic formation and PR-protein gene expression initiated by ROS generation is a novel effect of this particular monosaccharide in plants, and thus d-allose might be a candidate agent to test for reduction of disease development in rice (Kano *et al.*, 2010).

ROS are generated by NADPH oxidase in defence responses in many plants (Doke, 1985; Yin *et al.*, 2000; Ono *et al.*, 2001; Torres *et al.*, 2005; Sagi and Fluhr, 2006), and rice *Osrboh* genes encoding an NADPH oxidase have been identified (Wong *et al.*, 2007). Shimamoto’s group (Kawasaki *et al.*, 1999; Wong *et al.*, 2007) showed that ROS functions in a regulatory mechanism by forming a multiprotein complex with OsrbohB. In this study, the involvement of rbohs in the d-allose-induced rice defence induction was also identified because treatment with DPI, an NADPH oxidase inhibitor (Kawasaki *et al.*, 1999), inhibited ROS accumulation in d-allose-treated leaves, and the expression of the *OsrbohC* gene was typically induced after d-allose treatment. It was then found that the *OsrbohC*-overexpressing plants were more sensitive to d-allose for induction of ROS accumulation. Overexpression of *OsrbohC* did not result in constitutive ROS production, perhaps because rboh is known to require post-transcriptional regulation for ROS generation that is induced only after a trigger by various stresses (Doke, 1985; Kawasaki *et al.*, 1999; Sagi and Fluhr, 2006; Wong *et al.*, 2007). For example, Ca^2+^ influx into the cytoplasm and changes in protein phosphorylation are implicated in activating rboh (Sagi and Fluhr, 2006), and many other proteins including small GTPase Rac/Rop are involved in regulating the OsrbohB complex (Wong *et al.*, 2007; Nakashima *et al.*, 2008). Although candidate proteins for the putative complex that includes OsrbohC are not clear yet, Shimamoto’s group (Wong *et al.*, 2007) reported that Rac2, Rac6, and Rac7 could directly interact with OsrbohC in their yeast two-hybrid system, perhaps indicating the involvement of Rac in OsrbohC activation after d-allose treatment.

A6P was detected in rice leaves after d-allose treatment. Although HXK has been known as the first enzyme in the hexose assimilation pathway (Jang *et al.*, 1997; Rolland *et al.*, 2006), the presence of plant enzymes responsible for the phosphorylation of d-allose has never been reported. However, some HXKs from yeast and *T. caldophilus* have the potential to phosphorylate various aldohexoses including d-allose (Chenault *et al.*, 1997; Bae *et al.*, 2005), and it was established that rice HXKs, known to possess a glucose kinase function (Cho *et al.*, 2009), can also catalyse d-allose phosphorylation. Rice OsHXK5 and OsHXK6 were selected to test as the target HXKs because these rice HXKs are considered to be comparable in function with *Arabidopsis* AtHXK1 (Cho *et al.*, 2009), and a loss-of-function mutant of AtHXK1 [*glucose-insensitive2* (*gin2*) mutant] had a d-allose-insensitive phenotype for inhibition of vegetative growth of *Arabidopsis* seedlings (Fukumoto *et al*., 2011, 2013).

Reduced conversion of d-allose to A6P by HXK inhibition and a modification of d-allose at carbon 6 (6-deoxy-d-allose) to block phosphorylation prevented any defence responses, indicating the importance of d-allose conversion to A6P in d-allose signal transduction. Thus, overexpression of *E. coli AlsK* was tested, which is more efficient for A6P production than OsHXK5 or OsHXK6 but less efficient for G6P production from d-glucose, and the conversion of A6P from d-allose increased in *AlsK*-overexpressng rice, as did sensitivity to d-allose for inducing defence responses including ROS induction, lesion mimic formation, PR-protein gene expression, and disease resistance against *Xoo*. *Escherichia coli AlsI* was also overexpressed to convert the accumulated A6P to P6P; defence induction was reduced, further elucidating the importance of A6P in d-allose signal transduction. Together these results indicate that HXK is the initial contact site for d-allose in rice cells, and the conversion of d-allose to A6P is essential for the defence responses in rice (Supplementary Fig. S9 at *JXB* online).

Many monosaccharides play a role in signal transduction for cellular functions through their phosphorylation during sugar metabolism (e.g. Rolland *et al.*, 2006; Chu *et al.*, 2010). During glycolysis, phosphorylated d-glucose G6P can be converted to F6P by G6P isomerase and converted to 6-phosphogluconolactone by G6PDH in the pentose-phosphate cycle (Rolland *et al.*, 2006). The pentose-phosphate cycle, which generates NADPH and is related to redox regulations (Kruger and von Schaewen, 2003; Wakao and Benning, 2005; Ratcliffe and Shachar-Hill, 2006), is also considered to be involved in plant defence systems because inhibiting G6PDH reduces ROS generation induced by elicitor treatment (Pugin *et al.*, 1997) and because cytosolic overexpression of the P2 type of G6PDH leads to the induction of disease resistance with ROS generation via NADPH oxidase (Scharte *et al.*, 2009). G6PDH also seems to be involved in various cellular regulations via post-transcriptional modifications (Bulteau *et al.*, 2001; Gupte *et al.*, 2009), but the exact roles of this enzyme other than as the initial enzyme of the pentose-phosphate pathway are not clear. Plant G6PDH can be regulated by redox balance (Wakao and Benning, 2005), and a complex formation of the P0 type of G6PDH leads to a change in localization of other P1-type G6PDHs (Meyer *et al.*, 2011).

In this study, it was found that a G6PDH inhibitor reduced d-allose-derived ROS generation, the defence responses of a *Tos17*-inserted mutant of *OsG6PDH1* were less sensitive to d-allose, and lines complemented with *OsG6PDH1* recovered full sensitivity to d-allose. These results revealed that cytosolic OsG6PDH1 is involved in d-allose signal transduction to induce defence responses (Supplementary Fig. S9 at *JXB* online). Gene expression of plastidic isoforms of the P2 type of *OsG6PDH3* and the P0 type of *OsG6PDH5* were also induced by d-allose treatment. Similar to the case of *OsG6PDH1* at 12h after d-allose treatment, *OsG6PDH3* expression was induced more by d-allose than by d-glucose. Although involvement of these plastidic rice G6PDHs in ROS generation caused by plasma membrane-localized NADPH oxidase is not known yet, further study will be fascinating because formation of a protein complex with G6PDH isoforms was reported to lead to a change in the localization of other G6PDH isoforms (Meyer *et al.*, 2011), and a plastidic type of G6PDH was reported to be involved in NADPH oxidase-dependent ROS generation induced by an elicitor in tobacco (Asai *et al.*, 2011). Multiple reports describe NADPH derived from G6PDH reaction for ROS generation (e.g. Scharte *et al.*, 2009; Gutpe *et al*., 2009; Spencer *et al.*, 2011), but recombinant OsG6PDH1 did not use A6P as a substrate, indicating that d-allose-triggered ROS generation by NADPH oxidase is probably not caused by simple activation of OsG6PDH1 reaction by HXK-derived A6P. Interestingly, G6PDH activity in d-allose-treated leaves increased at 6h after treatment and the level was maintained even after 24h; however, G6PDH activity in d-glucose-treated leaves had increased by 6h after the treatment and returned to the control level by 12h. Since plant G6PDH is an unstable protein (Wakao and Benning, 2005), it was hypothesized that the G6PDH activity after 12h in the d-allose-treated leaves may be due to A6P stabilizing G6PDH. Various phosphorylated monosaccharides can interact with G6PDH (Scott and Tatum, 1971), and these substrates and cofactors also help stabilize the G6PDH (Puchkaev *et al.*, 2002; Wang and Engel, 2009). Phosphorylated monosaccharides are known to increase protein stability or activation. For example, glycogen synthase can bind G6P, which is not a substrate or cofactor of this enzyme, thus rearranging the subunit interface and facilitating catalysis by freeing the active site (Baskaran *et al.*, 2010). G6PDH can also be regulated by phosphorylation (Bulteau *et al.*, 2001; Gupte *et al.*, 2009), and protein kinases are post-transcriptionally involved as regulatory proteins of G6PDH (Gupte *et al.*, 2009; Santo *et al.*, 2012). A6P might interact with regulatory proteins other than G6PDH to activate or maintain the stability of G6PDH to generate NADPH by G6P usage as well. This body of information indicates that the exact targets and functions of A6P on the d-allose effects described here will provide more insight into novel roles for phosphorylated sugars in the future.

## Supplementary data

Supplementary data are available at *JXB* online.

Figure S1. Fisher projections of various monosaccharide structures used in this study.

Figure S2. Effect of d-allose concentration on *Xoo* growth in liquid culture.

Figure S3. OsrbohC overexpression in rice.

Figure S4. Metabolic pathway of d-allose in *Escherichia coli*.

Figure S5. *E. coli* d-allose kinase (AlsK) overexpression in rice.

Figure S6. *E. coli* d-allose 6-phosphate isomerase (AlsI) overexpression in rice.

Figure S7. Characterization of rice *G6PDH* genes.

Figure S8. Characterization of *Tos17* mutants for *OsG6PDH1* and its complementation.

Figure S9. Schematic model of d-allose signal transduction for induction of rice resistance to *Xoo.*

Table S1. Primers used in this study.

Table S2. Enzymatic profiles for OsG6PDH1- and OsG6PDH2-recombinant proteins using NADP+ as a kinetic parameter.

Table S3. Property summary for OsG6PDH1- and OsG6PDH2-recombinant proteins.

Supplementary Data
