# Supplementary material for: The rare sugar d-allose acts as a triggering molecule of rice defence via ROS generation
Source: J Exp Bot. 2013 Sep 7;64(16):4939–51. doi: 10.1093/jxb/ert282 (PMC3830479; doi:10.1093/jxb/ert282)
Supplement: Supplementary Data [file supp_64_16_4939__index.html]

The rare sugar d-allose acts as a triggering molecule of rice defence via ROS generation — The rare sugar d-allose acts as a triggering molecule of rice defence via ROS generation — The rare sugar d-allose acts as a triggering molecule of rice defence via ROS generation — Supplementary Data 

# The rare sugar d-allose acts as a triggering molecule of rice defence via ROS generation

## Supplementary Data

Data files

**Files in this Data Supplement:**

- Supplementary Data - Supplementary Data
