# Supplementary material for: The rare sugar d-allose acts as a triggering molecule of rice defence via ROS generation
Source: J Exp Bot. 2013 Sep 7;64(16):4939–51. doi: 10.1093/jxb/ert282 (PMC3830479; doi:10.1093/jxb/ert282)
Supplement: Supplementary Data [file supp_ert282_jexbot102319_file001.pdf]

Supplemental Figures and Tables

Figs. S1-S9
Tables S1-S3

RESEARCH PAPER:

Rare sugar D-allose acts as a triggering molecule of rice defense via ROS generation

Akihito Kano[1], Takeshi Fukumoto[1], Kouhei Ohtani[1], Akihide Yoshihara[1], Toshiaki Ohara[2], Shigeyuki Tajima[1], Ken Izumori[1], Keiji Tanaka[2], Takeo Ohkouchi[2], Yutaka Ishida[3], Yoko Nishizawa[4], Kazuya Ichimura[1], Yasuomi Tada[1], Kenji Gomi[1] and Kazuya Akimitsu[1,*]

[1]Faculty of Agriculture, Rare Sugar Research Center, and Gene Research Center, Kagawa University, Miki, Kagawa 761-0795, Japan; [2]Mitsui Chemicals Agro Inc., Yasu, Shiga 520-2342, Japan; [3]Shikoku Research Institute Inc., Yashima-nishi, Takamatsu 761-0192, Japan; and [4]National Institute of Agrobiological Sciences, Tsukuba 305-8602, Japan.

[*]To whom correspondence should be addressed: E-mail: kazuya@ag.kagawa-u.ac.jp
+81-87-891-3131 (Tel); +81-87-891-3021 (Fax)

Supplemental figure and table legends

Figure and table legends

Fig. S1. Fisher projections of various monosaccharide structures used in this study.

Four common sugars (D-glucose, D-fructose, D-galactose, and D-mannose), nine rare sugars, and their derivatives (D-glucose 6-phosphate, D-allose 6-phosphate, 6-deoxy D-allose) are shown.

Fig. S2. Effect of D-allose concentration on *Xoo* growth in liquid culture.

Mean OD600 (± SE) of cultures amended with various D-allose concentrations at selected times during three independent experiments. (E)-2-Hexenal served as the control possessing antimicrobial activity (Gomi et al., 2010).

Fig. S3. OsrbohC overexpression in rice.

(A) Construct of OsrbohC overexpression vector in pBI333-EN4.

(B) Second generation of lines 11 and 28, selected from multiple transgenic rice plants expressing OsrbohC, and WT. No visible phenotype resulted with OsrbohC overexpression.

(C) Excess OsrbohC did not change sensitivity to *Xoo* with or without 50 mM D-glucose (D-Glc).

Fig. S4. Metabolic pathway of D-allose in *Escherichia coli*.

Metabolic pathway of D-allose in *E. coli* described by Kim et al. (1997) was shown.

Fig. S5. *E. coli* D-allose kinase (AlsK) overexpression in rice.

(A) Construct of AlsK overexpression vector in pBI333-EN4.

(B) Second generation of independent lines 6 and 21, selected from multiple transgenic rice plants expressing AlsK, and WT. No visible phenotype resulted with AlsK

overexpression.

(C) Excess AlsK did not change sensitivity to *Xoo* with or without 50 mM D-glucose (D-Glc).

(D-E) D-Glucose (D) or D-glucose 6-phosphate (G6P) (E) content in leaves from WT and AlsK-overexpressing lines, relative (± SE, n = 3) to WT, as assessed by HPLC,

(F-K) HPLC detection of ABEE-labeled monosaccharides in extracts from leaves from 24-h D-allose treatment. Each panel on right shows expanded detail of left panel from 1 to 25 min. Absolute value of D-glucose in leaves in panel D was calculated as 2.545 $\mu g \cdot gFW^{-1}$. Abbreviations used: D-allose, D-All; D-allose 6-phosphate, A6P; D-glucose, D-Glc; and D-glucose 6-phosphate, G6P.

Fig. S6. *E. coli* D-allose 6-phosphate isomerase (AlsI) overexpression in rice.

(A) Construct of AlsI overexpression vector in pBI333-EN4.

(B) Second generation of independent lines 13 and 14 selected from multiple transgenic rice plants expressing AlsI and WT. No visible phenotype resulted with AlsI overexpression .

(C-H) HPLC detection of ABEE-labeled monosaccharides in extracts from leaves treated with D-allose for 24 h. Each panel on right shows expanded detail of left panel from 1 to 25 min.

Abbreviations used: D-allose, D-All; D-allose 6-phosphate, A6P; D-glucose, D-Glc; and D-glucose 6-phosphate, G6P.

Fig. S7. Characterization of rice *G6PDH*s.

(A) qRT-PCR analysis of *OsG6PDH2*–*5* gene (accession nos. are in panel B) expression in leaves at 0 to 24 h after treatment with 5 mM D-allose (D-All) or

D-glucose (D-Glc), relative to control (no sugar) (Con) (± SE, *n* = 4).

(B) Phylogenetic analysis of *OsG6PDH1–5* genes with Arabidopsis *G6PD* genes using Clustal W at the DDBJ (http://www.ddbj.nig.ac.jp/) and 1,000 bootstraps. Respective clades were categorized according to those for Arabidopsis *G6PD* genes (Wakao et al., 2005).

(C) Subcellular localization of GFP-tagged OsG6PDH1 and OsG6PDH2 in plant cells. Epidermal layers of tobacco leaves were bombarded with particles coated with constructs to express GFP alone (upper), OsG6PDH1-GFP (middle), and OsG6PDH2-GFP (lower).

Fig. S8. Characterization of *Tos17* mutants for *OsG6PDH1* and its complementation.

(A) Exon and intron organization of *OsG6PDH1* and various *Tos17* insertion sites. Solid boxes and lines indicate exons and introns; triangles indicate position of *Tos17* insertion. Of 10 lines with *Tos17*-inserted *OsG6PDH1* mutations at the protein-coding region of exon 10 and 11 found in the rice *Tos17* mutant database (Fig. S8A) (Miyao et al., 2003), lines NC0350 and NC0573 have morphologically abnormal phenotypes, and the insertion of *Tos17* did not match the sites recorded for lines NC0293, NC0320, NC0395, and NC0555. For lines NC0322, NC0695, and NC2550 examined by PCR for *Tos17* insertion, no insertion was found at the target site in exon 10 of *OsG6PDH1* (data not shown). Among mutant lines, *Tos17* was inserted at the target site in exon 11 in NC8489, which we then examined further.

(B) Construct of *OsG6PDH1* complementation vector in pBI333-EN4 consists of a 4099-bp DNA fragment with *OsG6PDH1* promoter region (2496 bp) connected to entire cDNA (1551 bp) of *OsG6PDH1* gene with 42-bp 3'-untranslated region by 10-bp

linker.

Fig. S9. Schematic model of D-allose signal transduction for induction of rice resistance to *Xoo*. Conversion of D-allose to A6P by HXK induces ROS generation by OsrbohC following PR-gene expression, lesion mimic formation, and resistance to *Xoo* with involvement of G6PDH1 in rice.

Table S1. Primers used in this study.

F, forward direction; R, reverse direction. Extra nucleotides attached to introduce restriction sites are underlined.

Table S2. Enzymatic profiles for OsG6PDH1- and OsG6PDH2-recombinant proteins using NADP+ as a kinetic parameter.

Kinetic parameters were determined using G6PDH-coupled assay for G6P (Wakao and Benning, 2005).

Table S3. Property summary for OsG6PDH1- and OsG6PDH2-recombinant proteins.

[a]Kinetic parameters were determined using G6PDH-coupled assay for A6P with a maximum concentration of 5 mM (Wakao and Benning, 2005). ND: not detected.

[b]G6PDH activity with G6P with 10 mM DTT.

Experimental Procedures for supplemental results

Antimicrobial activity analysis

Xoo was cultured in YT (0.5% (w/v) Bacto-Yeast Extract (Becton, Dickinson & Co.), 1% (w/v) Bacto-Tryptone (Becton, Dickinson & Co.), pH 6.8) liquid medium

containing 1, 5 or 10 mM of D-allose or 100 μM (E)-2-hexenal. (E)-2-Hexenal was dissolved in DMSO and used as the control possessing antimicrobial activity by the method previously described (Gomi *et al*., 2010).

Transient localization assay

G6PDH1- or G6PDH2-GFP fusion proteins were expressed using a particle gun-mediated DNA delivery to tobacco epidermal cells and imaged using epifluorescence system DP70-SET-A, differential interference contrast optics and an Olympus BX51 microscope (Olympus, Tokyo, Japan). After bombardment, tissues were incubated for 24 h at 24˚C in the dark; ca. 100 cells/construct were examined for GFP localization in at least three independent experiments. All methods are as described by Yamasaki and Akimitsu (2007).

Phylogenetic tree analysis

Phylogenetic tree was constructed using both rice (*OsG6PDH*) and Arabidopsis (*G6PD*) (Wakao *et al.*, 2008) sequences were used for Clustal W (Thompson *et al*., 1994) analyses at the DNA Data Bank of Japan (http://www.ddbj.nig.ac.jp/) using 1,000 bootstraps. Consensus tree was visualized in TREEVIEW (Page, 1996).

Genotypic determination of homozygote for Tos17 insertion

Total DNA from leaves of WT and *Tos17* mutant lines were isolated using NecleoSpin plant II (MACHEREY-NAGEL, Hoerdt, France) and manufacturer's instructions. PCR was run using KOD DNA polymerase (TOYOBO, Osaka, Japan) in a thermal cycler with initial PCR activation at 94˚C for 2 min followed by 30 cycles of 3-step cycling (denaturation at 98˚C for 10 sec annealing at 60˚C for 30 sec, and extension at 68˚C for 1 min) with gene-specific primers used for PCR (Table S1).

Recombinant G6PDH assays

Activity of recombinant rice G6PDHs was measured spectrophotometrically (340 nm) at 25°C by detecting NADP reduction via G6PDH reaction, which is coupled with G6P production, as described by Wakao and Benning (2005) as described in the main text. To determine *K*i NADPH of G6PDH, activity was examined with 0.01 to 0.1 mM NADPH.

References specific for supplemental information

Page RDM. 1996. TREEVIEW: An application to display phylogenetic trees on personal computers. *Computer Applications in the Biosciences* **12**, 357-358.

Thompson JD, Higgins DS, Gibson TJ. 1994. CLUSTAL W: improving the sensitivity of progressive multiple sequence alignment through sequence weighting, position-specific gap penalties and weight matrix choice. *Nucleic Acids Research* **22**, 4673-4680.

Yamasaki Y, Akimitsu K. 2007. *In situ* localization of gene transcriptions for monoterpene synthesis in irregular parenchymic cells surrounding the secretory cavities in rough lemon (*Citrus jambhiri*). Journal of Plant Physiology **164**, 1436-1448.

Table S1. Primers used in this study.

	Direction	Primer sequences (5'-3')
Construction of overexpression vectors		
AlsK	F	GCTCTAGAGCATGCAAAAACAGCATAACGTC
	R	GGGGTACCCCTCATGGGGCTTTAGCACAGAAC
AlsI	F	GCTCTAGAGCATGAAAAAGATTGCATTTGGCTGTG
	R	GGGGTACCCCTCAATTTCTCCGCTGCTCTATTG
OsrbohC	F	GCTCTAGAGCTTGGTGGTGGGCTAGCTG
	R	GGGGTACCCCCTCTATGCTTCTCGCCCTTG
Tos17 analysis by genome PCR		
G6PDH1-F	F	CCTTTCATTCTCAAGGCTGG
G6PDH1-R	R	CCCGGCAAAAACCAATAGTA
Tos17-F	F	ATTGTTAGGTTGCAAGTTAGTTAAGA
Construction of G6PDH1complimentation vectors		
OsG6PDH1 promoter region	F	ACATGCATGCATGTTGGCCGACGGTGAAGTC
	R	GCTCTAGAGCGATGAACTGTATGGTCTCTTCCTTTG
OsG6PDH1 translated region	F	GCTCTAGAGCATGTCAGGAGGATCTGGTGATTC
	R	GCTCTAGAGCAACCGGCATCTAGACAGAGC
Transgenic analysis by RT-PCR		
AlsK	F	ATGCAAAAACAGCATAACGTC
	R	GTATAATTGCGGGACTCTAATC
AlsI	F	ATGAAAAAGATTGCATTTGGCTGTG
	R	GTATAATTGCGGGACTCTAATC
OsrbohC	F	GTGATCTTTACCCGCCAATC
	R	GTATAATTGCGGGACTCTAATC
OsG6PDH1	F	ATGTCAGGAGGATCTGGTGATTC
	R	CTAGAATTTTGAAAGGGTTGGAG
Actin	F	CCTGGAATCCATGAGACCAC
	R	ACACCAACAATCCCAAACAGAG
qRT-PCR analysis		
PBZ1	F	GTGGTTGTGTTTATGTGCCTTTCTATG
	R	ACTTGCCTCTCTTTATTCACCCATTG
PR1b	F	AGTGTCTGATCCACGCCTTC
	R	ACCTGAAACAGAAAGAAACAGAGG
POX22.3	F	GGATGCGTTCGTTGCTGGAAG
	R	CAACACCACCGTACCTATACTTGTG
β-1,3-glucanase	F	ACGAGACGGAGAGGCACTTC
	R	TCGATCCCTTCTCAGAACAATCTTC
IAI	F	TGCTGTCATATATCTGTGATCTGTGTG
	R	CCTTCACTCAGACAACGGCTTATTG
Proteinase inhibitor	F	GTGTGTTCTAGCTTGTTCGTATTCG
	R	CACGCATACCAACATCAAACCAC
Lipoxygenase	F	CATCTGTTTGGAGTGCTCATC
	R	TATACGGTGTCCTGTACTTACTAC
Chitinase	F	ACGGCATCACCAACATCAT
	R	GTAGCCTGTGCCGAGCAT
TLP	F	GCCCTGTCGTGCACCCT
	R	CAGGTAAGCGTCGGGGCA
OsrbohA	F	TGCAGCATGTCTTATGCAGTC
	R	GCCTTGTGACCTTCCTATGTG
OsrbohB	F	ATGGTTCCGACGTAACAAGC
	R	ACGTTCCATGGACAAAGAGC
OsrbohC	F	CAAGGGCGAGAAGCATAGAG
	R	CCAGATGATCCCCAAAACTC
OsrbohD	F	CAACGACTCGGTTCCATTTC
	R	TGAAAGAGTTGCTAACCGATGC
OsG6PDH1	F	CCAATGCAGGCTCTGTCTAG
	R	TTCCAAGATCACCAACATTACAG
OsG6PDH2	F	ATTTTCACGATAACGCCTGAC
	R	GTCGAAACATGCACTTTTATTTC
OsG6PDH3	F	CTTTTGCCATTGCATTTCTG
	R	GGCCTTGCAACCTAAGCTAAC
OsG6PDH4	F	TCGATCGGAGTCGCTGTTAC
	R	AATTTCGTCCCTGAGCAGTC

OsG6PDH5	F	TGGCCTAATTGCTCCAAATG
	R	GGAACCATTTCTATTTTTGTTGC
Actin	F	GAGTATGATGAGTCGGGTCCAG
	R	ACACCAACAATCCCAAACAGAG
Prodution of recombinant protein		
OsHXK5	F	GCGAGCTCGCATGGCGGCGGTGATCGAG
	R	CCTCGAGGCTAGTCGATCTCGGCATACTGG
OsHXK6	F	CGGAATTCCGATGGCCGCCGCTGTGATC
	R	CCCTCGAGGGCTACTCGACGCTAGCATACTGG
AlsK	F	CGGAATTCCGATGCAAAAACAGCATAACGTC
	R	CCCTCGAGGGTCATGGGGCTTTAGCACAGAAC
OsG6PDH1	F	CGTCGACGATGTCAGGAGGATCTGGTGATTC
	R	CCAAGCTTGGCTAGAATTTTGAAAGGGTTGGAGG
OsG6PDH2	F	CGTCGACGATGTCAGGAGGATCTTCACCAAG
	R	CCAAGCTTGGCTATGCAAGGGTGGGTGGTATC
Transient localization assay		
OsG6PDH1	F	GTCGACCGTCGTCGTCTTCTTCTTCC
	R	GTCGACGAATTTTGAAAGGGTTGGAGG
OsG6PDH2	F	GTCGACATGTCAGGAGGATCTTCACCAA
	R	GTCGACAAGGGTGGGTGGTA

F, forward direction; R, reverse direction. Extra nucleotides attached to introduce restriction sites are underlined.

Table S2. Enzymatic profiles for OsG6PDH1- and OsG6PDH2-recombinant proteins using $NADP^+$ as a kinetic parameter.

Enzyme	*k*cat (s^{-1})	*Km* $NADP^+$ (M)	*k*cat/*K*m $NADP^+$ (M^{-1} s^{-1})	*K*i NADPH (M)
G6PDH1	3.04	6.49×10^{-5}	4.68×10^{4}	4.15×10^{-5}
G6PDH2	1.06	4.1×10^{-5}	2.59×10^{4}	3×10^{-5}

Kinetic parameters were determined using G6PDH-coupled assay for G6P (Wakao and Benning, 2005).

Table S3. Property summary for OsG6PDH1- and OsG6PDH2-recombinant proteins.

Enzyme	A6P[a]	Reduction[b]	Group
G6PDH1	ND	Insensitive	Cytosolic
G6PDH2	ND	Insensitive	Cytosolic

[a]Kinetic parameters were determined using G6PDH-coupled assay for A6P with a maximum concentration of 5 mM (Wakao and Benning, 2005). ND: not detected.
[b]G6PDH activity with G6P with 10 mM DTT.

Aldose

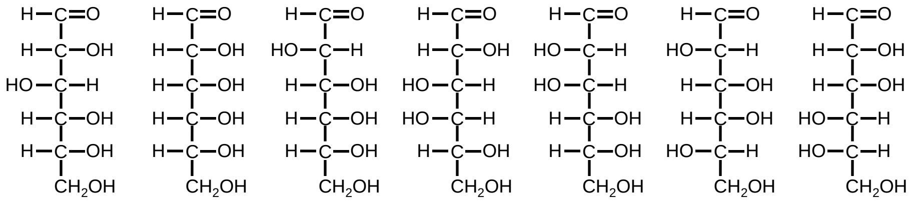

D-glucose D-allose D-altrose D-galactose D-mannose L-galactose L-mannose

Ketose

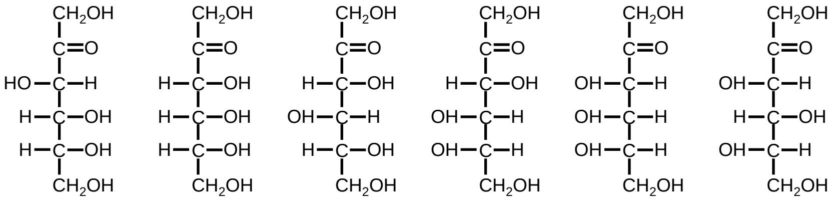

D-fructose D-psicose D-sorbose L-fructose L-psicose L-sorbose

Sugar derivative

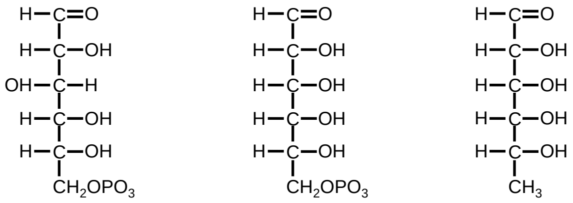

D-glucose 6-phosphate D-allose 6-phosphate 6-deoxy D-allose

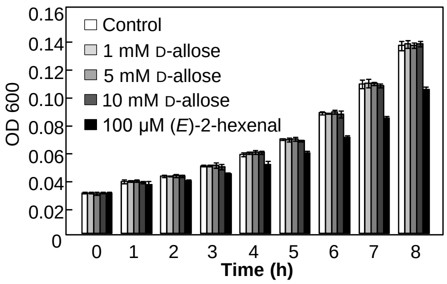
0.16
0.14
0.12
0.10
0.08
0.06
0.04
0.02
0
OD 600
Control
1 mM D-allose
5 mM D-allose
10 mM D-allose
100 μM (E)-2-hexenal
0
1
2
3
4
5
6
7
8
Time (h)

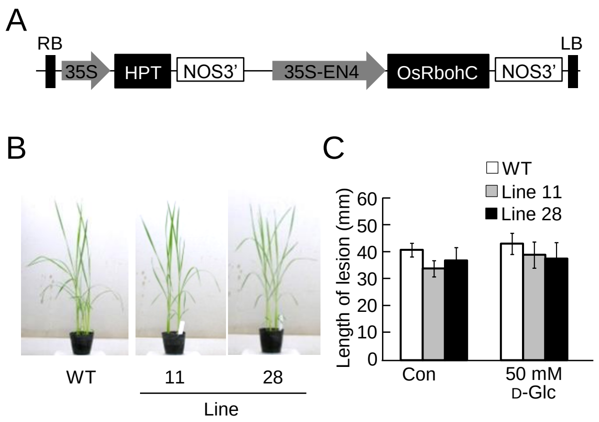
A
RB
LB
35S
HPT
NOS3'
35S-EN4
OsRbohC
NOS3'
B
WT
11
28
Line
C
WT
Line 11
Line 28
Length of lesion (mm)
60
50
40
30
20
10
0
Con
50 mM
D-Glc

Kano et al Fig. S4

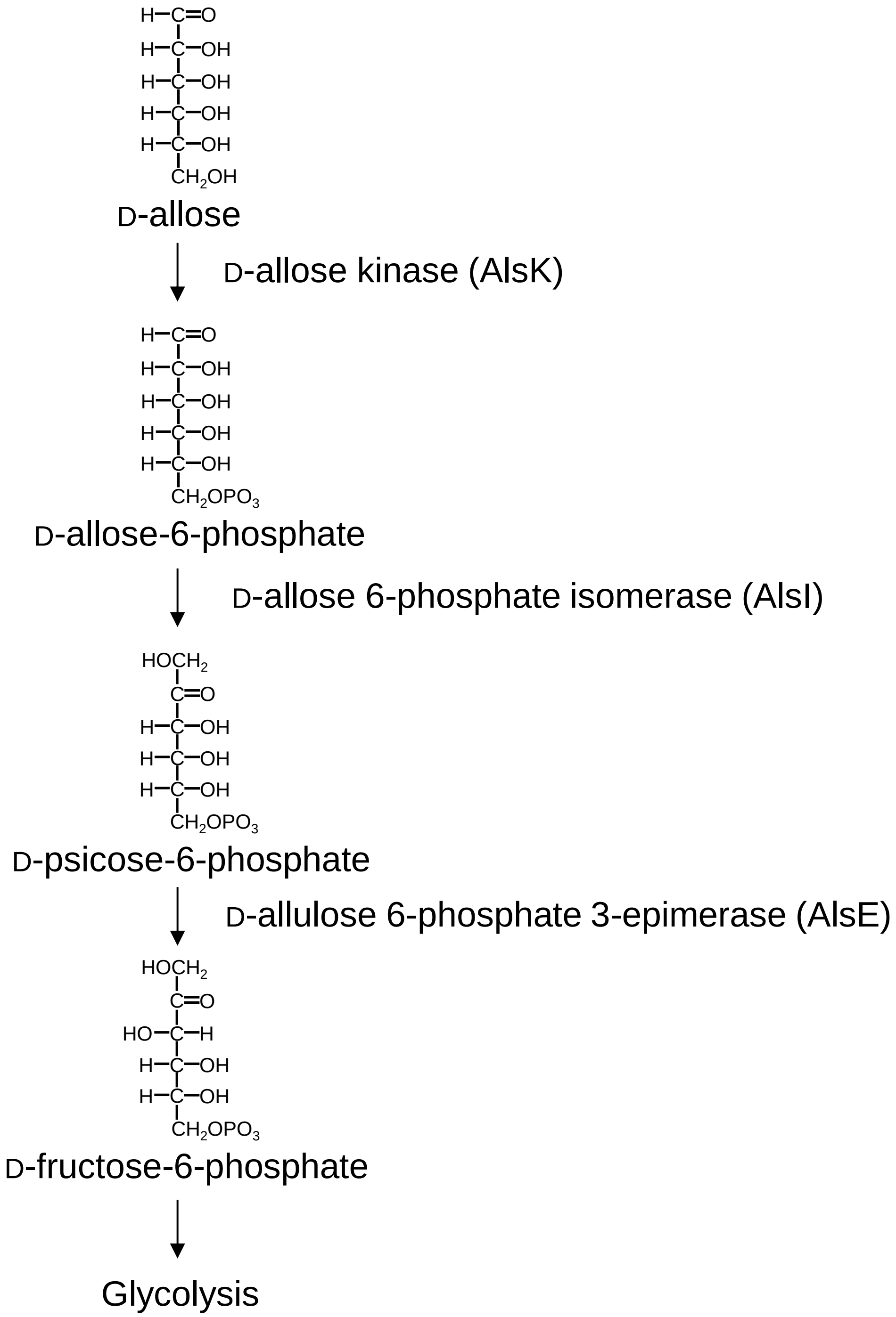

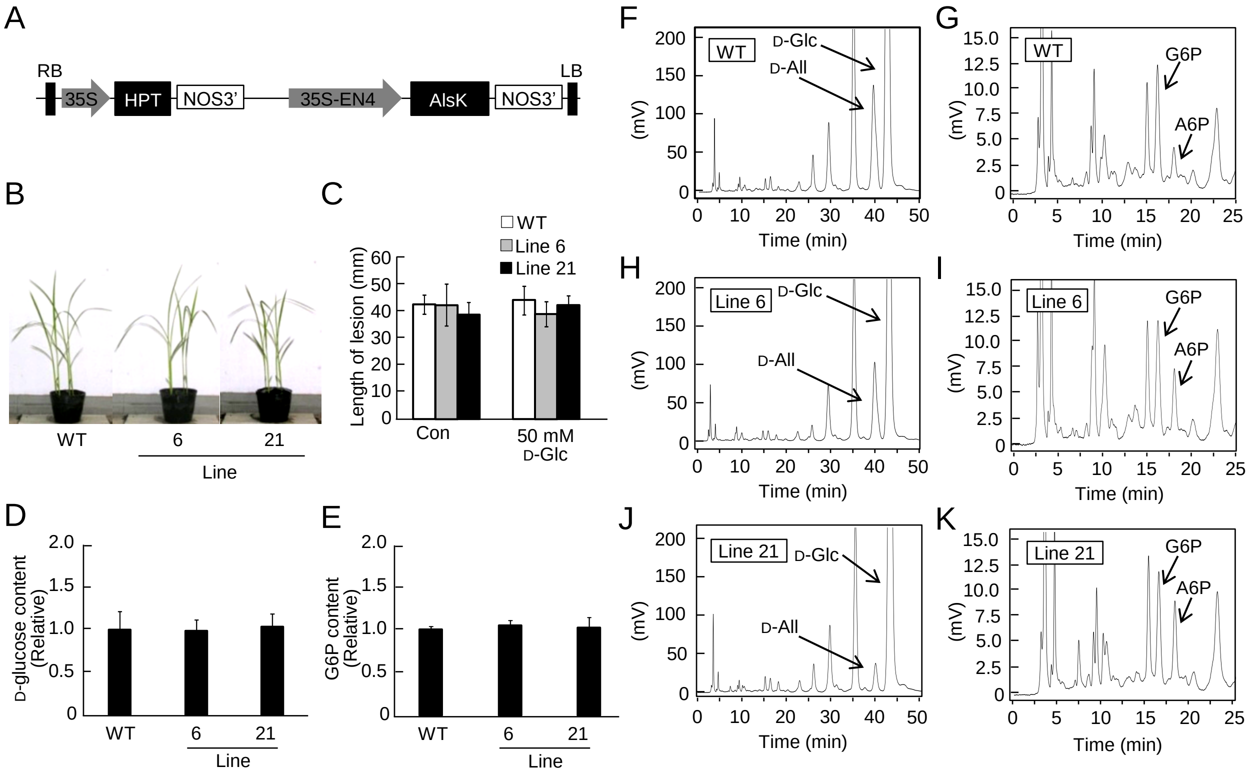
A
RB
35S
HPT
NOS3'
35S-EN4
AlsK
NOS3'
LB
B
WT
6
21
Line
C
WT
Line 6
Line 21
Length of lesion (mm)
Con
50 mM D-Glc
D
D-glucose content (Relative)
WT
6
21
Line
E
G6P content (Relative)
WT
6
21
Line
F
WT
D-Glc
D-All
(mV)
Time (min)
G
WT
G6P
A6P
(mV)
Time (min)
H
Line 6
D-Glc
D-All
(mV)
Time (min)
I
Line 6
G6P
A6P
(mV)
Time (min)
J
Line 21
D-Glc
D-All
(mV)
Time (min)
K
Line 21
G6P
A6P
(mV)
Time (min)

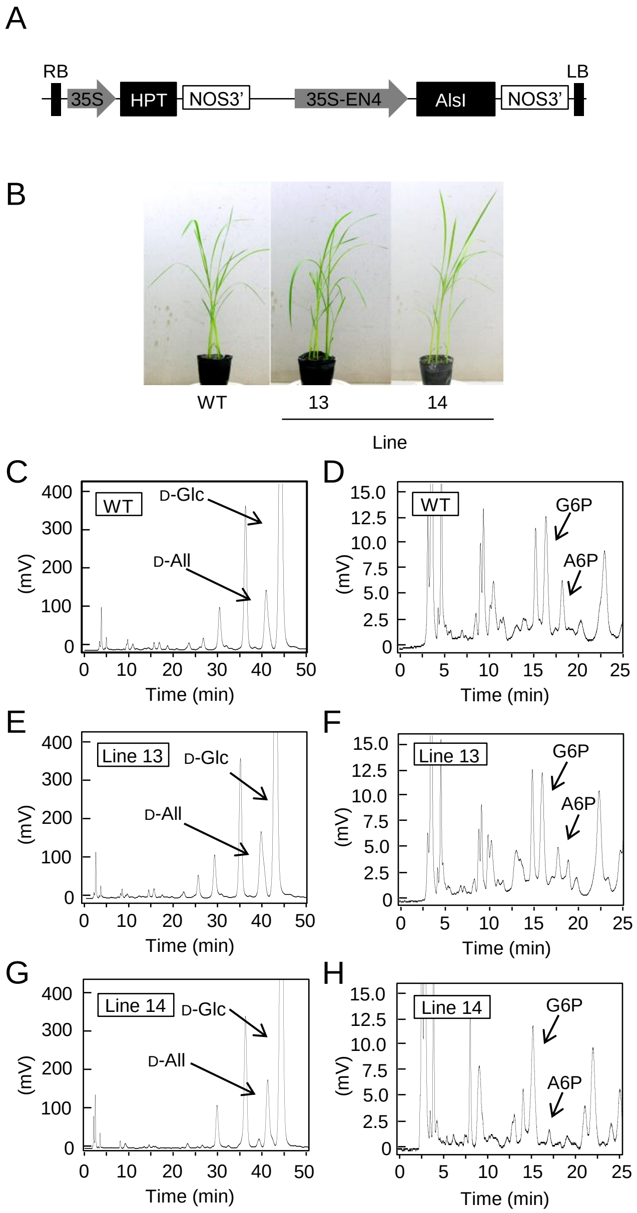
A
RB
35S
HPT
NOS3'
35S-EN4
AlsI
NOS3'
LB
B
WT
13
14
Line
C
WT
D-Glc
D-All
(mV)
Time (min)
D
WT
G6P
A6P
(mV)
Time (min)
E
Line 13
D-Glc
D-All
(mV)
Time (min)
F
Line 13
G6P
A6P
(mV)
Time (min)
G
Line 14
D-Glc
D-All
(mV)
Time (min)
H
Line 14
G6P
A6P
(mV)
Time (min)

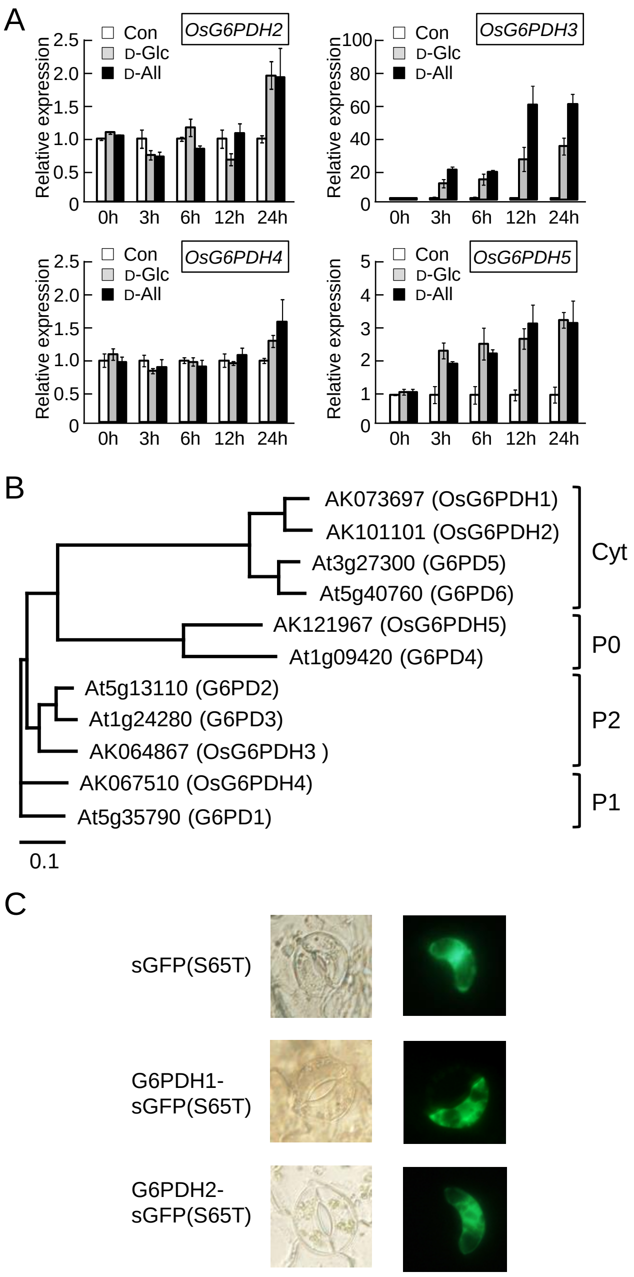
A
Con
D-Glc
D-All
OsG6PDH2
OsG6PDH3
OsG6PDH4
OsG6PDH5
Relative expression
0h 3h 6h 12h 24h
B
AK073697 (OsG6PDH1)
AK101101 (OsG6PDH2)
At3g27300 (G6PD5)
At5g40760 (G6PD6)
AK121967 (OsG6PDH5)
At1g09420 (G6PD4)
At5g13110 (G6PD2)
At1g24280 (G6PD3)
AK064867 (OsG6PDH3)
AK067510 (OsG6PDH4)
At5g35790 (G6PD1)
Cyt
P0
P2
P1
0.1
C
sGFP(S65T)
G6PDH1-
sGFP(S65T)
G6PDH2-
sGFP(S65T)

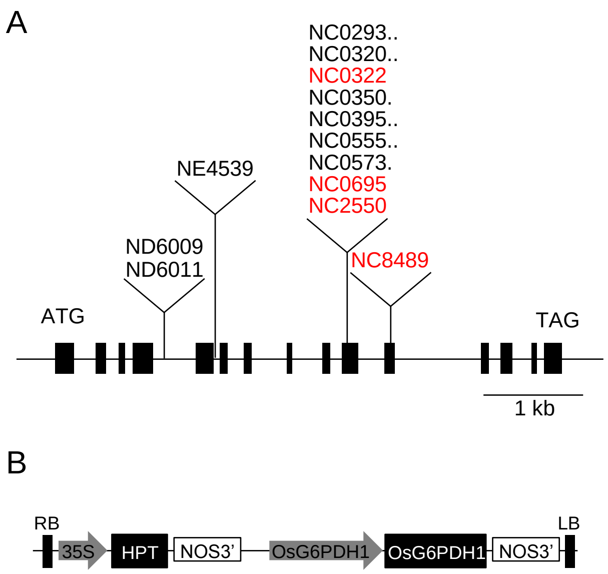
A
NC0293..
NC0320..
NC0322
NC0350.
NC0395..
NC0555..
NC0573.
NC0695
NC2550
NE4539
ND6009
ND6011
NC8489
ATG
TAG
1 kb
B
RB
LB
35S
HPT
NOS3'
OsG6PDH1
OsG6PDH1
NOS3'

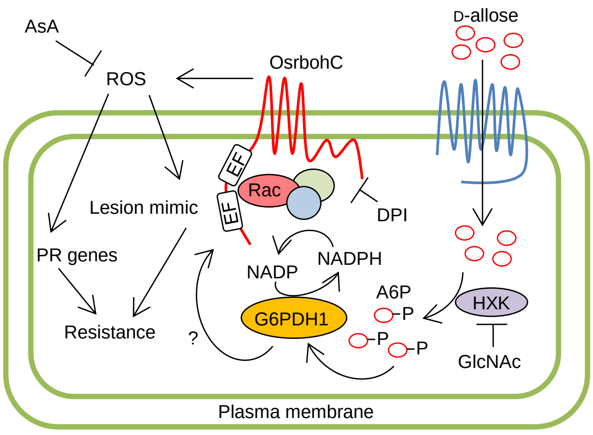
D-allose
AsA
ROS
OsrbohC
EF
EF
Rac
DPI
Lesion mimic
PR genes
Resistance
NADP
NADPH
A6P
P
P
P
HXK
G6PDH1
?
GlcNAc
Plasma membrane
